# A 3,2-Hydroxypyridinone-based Decorporation Agent that Removes Uranium from Bones In Vivo

**DOI:** 10.1038/s41467-019-10276-z

**Published:** 2019-06-25

**Authors:** Xiaomei Wang, Xing Dai, Cen Shi, Jianmei Wan, Mark A. Silver, Linjuan Zhang, Lanhua Chen, Xuan Yi, Bizheng Chen, Duo Zhang, Kai Yang, Juan Diwu, Jianqiang Wang, Yujie Xu, Ruhong Zhou, Zhifang Chai, Shuao Wang

**Affiliations:** 1 State Key Laboratory of Radiation Medicine and Protection, School for Radiological and Interdisciplinary Sciences (RAD-X) and Collaborative Innovation Center of Radiation Medicine of Jiangsu Higher Education Institutions, Soochow University, Suzhou, 215123 China; 2 Shanghai Institute of Applied Physics and Key Laboratory of Nuclear Radiation and Nuclear Energy Technology, University of Chinese Academy of Sciences, Shanghai, 201800 China; 3 Computational Biology Center, IBM Thomas J Watson Research Center, Yorktown Heights, NY 13 10598; Department of Chemistry, Columbia University, New York, NY 10027 United States

**Keywords:** Nuclear chemistry, Bone, Kidney, Bioinorganic chemistry

## Abstract

Searching for actinide decorporation agents with advantages of high decorporation efficiency, minimal biological toxicity, and high oral efficiency is crucial for nuclear safety and the sustainable development of nuclear energy. Removing actinides deposited in bones after intake is one of the most significant challenges remaining in this field because of the instantaneous formation of highly stable actinide phosphate complexes upon contact with hydroxyapatite. Here we report a hydroxypyridinone-based ligand (5LIO-1-Cm-3,2-HOPO) exhibiting stronger affinity for U(VI) compared with the reported tetradentate hydroxypyridinone ligands. This is further revealed by the first principles calculation analysis on bonding between the ligand and uranium. Both in vitro uranium removal assay and in vivo decorporation experiments with mice show that 5LIO-1-Cm-3,2-HOPO can remove uranium from kidneys and bones with high efficiencies, while the decorporation efficiency is nearly independent of the treatment time. Moreover, this ligand shows a high oral decorporation efficiency, making it attractive for practical applications.

## Abstract (web summary)

In vivo decorporation of U(VI) from bones is an unsolved challenge because of the formation of stable uranium phosphate complexes. Here, the authors develop a hydroxypyridonone-based ligand with strong uranium complexation and low cytotoxicity. They find this ligand effectively removes uranium from kidney and bones in mice, and is suitable for oral administration.

## Introduction

Continued development of in vivo decorporation agents that target key radionuclides from nuclear fuel cycle, especially actinides, is critical for public safety control during emergency responses to nuclear accidents and could serve as a preemptive measure to mitigate the damages from nuclear incidents linked to terrorism. Uranium is the most important naturally-occurring actinide and a crucial resource for nuclear power, which is also recognized as a global environmental contaminant due to its combined radio- and chemo-toxicities^1,2^. Although different routes that introduce uranium into the human body exist, e.g., through ingestion, inhalation, and wound related absorption, approximately two-thirds of uranium is subsequently eliminated from plasma and excreted through the kidney. However, the portion of uranium that remains is retained in deposits found in kidneys and bone tissues in the form of the hexavalent uranyl ion (UO_2_^2+^)^2^, leading to both acute and chronic renal damage, as well as a heightened risk of osteosarcoma and osteogenesis^3–8^. The latter hazard suffers from long-term and unsolved therapy challenges because of the instantaneous formation of highly insoluble uranyl phosphate complexes when uranium contacts calcium hydroxyapatite (HAP) in bones. A decorporation agent which can effectively remove uranium from these parts in humans is highly desirable.

Chelation therapy is the most effective treatment for uranium decorporation. A suitable multidentate ligand (i.e., chelator) is administered with the intent of selectively coordinating toxic metals and accelerating their excretion from the body, thereby reducing the toxicity via competitive formation of thermodynamically favorable and water-soluble molecular complexes^9^. A feasible chelation agent is chosen based on the criteria that the ligand displays a strong affinity towards uranyl ion and the coordination environment facilitates their removal^9^, while also exhibiting biocompatibility and low toxicity as a bare complexant. Diethylenetriamine pentaacetate calcium sodium salt (CaNa_3_-DTPA) and its zinc sodium salt (ZnNa_3_-DTPA) are currently the only Food and Drug Administration (FDA)-approved drugs for this purpose^10^. Additionally, NaHCO_3_ is also recommended by FDA in case of internal contamination of uranium. Although DTPA has been reported for insufficient removal of intracellular actinide deposits owing to its hydrophilic nature that limits its ability to cross the cell membranes^10–12^, Grémy et al. developed a liposomal DTPA with enhanced removal efficiency of Pu/Am in liver with either prophylactic or delayed treatment^13,14^. Moreover, although DTPA agents are effective with low-valent actinides such as Pu(IV) and Am(III), they show minimal effects on U(VI)^15–18^. Macrocycles have shown strong affinity for U(VI), however, decorporation assays aimed at their interaction with U(VI) in either animal or cellular assays are limited with relatively high toxicity^19^. Functional groups, such as catechol (CAM), terephthalamide (TAM), and hydroxypyridinone (HOPO) were evaluated for their decorporation efficiency with actinides^9,20^. Most of these ligands were found to be capable of removing significantly more U(VI) from the entire body and kidneys compared to CaNa_3_-DTPA under the same conditions. Despite that CAM(S), MeTAM, and 3,4,3-LI-CAM(C) ligands can significantly reduce U(VI) deposited in bone using a high ligand to U(VI) molar ratio, CAM(S) ligands were found to be severely toxic by introducing kidney damage, and both 4-LI-MeTAM and 5-LI-MeTAM caused serious damages to the kidneys, liver, and/or spleen^9^. Recently, a 3,2-hydroxypyridinone-grafted chitosan oligosaccharide nanoparticle (COS-HOPO) with low toxicity was shown to be effective for reactive oxygen species removal and can prevent uranium from depositing on kidney and bones by prophylactic administration^21^. In addition, a bidentate ligand 3-hydroxy-2-pyrrolidinone (HPD) was recently reported with a low cytotoxicity^22^. However, in these two works, the decorporation efficiency on removing uranium from kidney was limited, and more importantly no obvious removal effect from bones was observed by prompt intraperitoneal injection. Among all candidates reported, 5LIO-(Me-3,2-HOPO) and 3,4,3-LI(1,2-HOPO) are the two most promising chelators because of their high body removal efficiency for U(VI), low toxicities, and high oral activities^15,23,24^. The most important and probably the only drawback of these two ligands is their poor performances in removing uranium from bones at low ligand to metal molar ratio.

From previous studies, these HOPO ligands were intentionally designed to form intramolecular hydrogen bonds between the amide and hydroxide groups in order to be pre-deprotonated in the physiological pH range and could therefore more successfully coordinate U(VI) in a sense of kinetic control^23^. However, the formation of the intramolecular hydrogen bonding would partially offset the negative potential of the oxygen donors in the HOPO units, possibly leading to the decrease of the ligand’s affinity for U(VI). Therefore, in this work, we introduce a chelating agent (5LIO-1-Cm-3,2-HOPO) with an additional methyl group inserted between the amide and HOPO groups in order to significantly weaken these intramolecular hydrogen bonds. This ligand exhibits significantly elevated coordination capability towards U(VI), as demonstrated by potentiometric titration experiment, synchrotron radiation-based extended X-ray adsorption fine structure (EXAFS) analysis, and density functional theory (DFT) simulation. Ultimately, results of both in vitro and in vivo assays suggest that 5LIO-1-Cm-3,2-HOPO is a potential solution to the long-term challenge of efficiently removing uranium from bones.

## Results

### Ligand design and synthesis

**Fig. 1:**
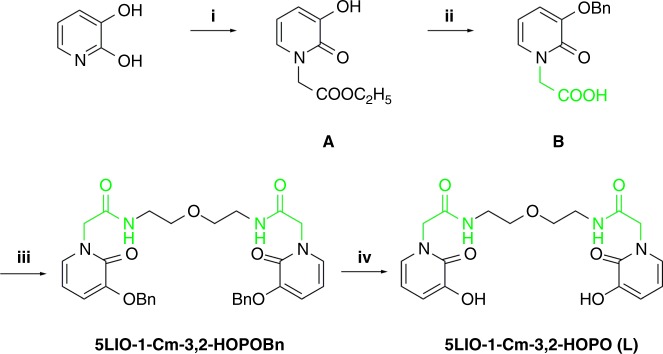
Synthesis procedure for 5LIO-1-Cm-3,2-HOPO. i: Br-CH_2_COOC_2_H_5_, 150 ^o^C, 24 h; ii: BnCl, NaOH, 80 ^o^C, 12 h; iii: NHS/EDC, 1,5-diamino-3-oxapentane, 30 ^o^C, 12 h; iv: Pd/C, H_2_, 30 ^o^C, 4 h

In order to weaken the intramolecular hydrogen bond formed between the amide and hydroxide groups found in many tetradentate HOPO ligands, a ligand {N,N′-[oxybis(ethane-2,1-diyl)]bis[2-(3-hydroxy-2-oxopyridin-1(2*H*)-yl) acetamide], denoted as 5LIO-1-Cm-3,2-HOPO, was designed by introducing the carboxylic group on the N site of pyridine ring. The distance between the amide and carbonyl group is increased by the addition of a methyl group. 5LIO-1-Cm-3,2-HOPO was obtained from a 4-step synthesis as illustrated in Fig. 1^25–27^. Commercially available 1,2-dihydro-2,3-pyridinediol was first alkylated at the nitrogen position of the pyridinone ring by reacting it with excess amounts of ethyl bromoacetate at 150 °C to yield the ester (product A). Next, the hydroxyl group in A was protected with a benzyl moiety, and the ester group was activated via hydrolysis to afford product B. 5LIO-1-Cm-3,2-HOPOBn was then obtained via amidation of the carboxylate group with the amine backbone. Benzyl protection was finally removed by 5% Pd/C to obtain 5LIO-1-Cm-3,2-HOPO with a total yield of 43%. The chemical shifts observed in the spectra of ^1^H NMR and ^13^C NMR all corroborate with the chemical structure of the molecules, and the results of elemental analysis and LC-MS are consistent with the formula of 5LIO-1-Cm-3,2-HOPO (Supplementary Figure 1). In the FTIR spectra, the pattern of 5LIO-1-Cm-3,2-HOPO contains peaks at 2947 cm^−1^ and 1080 cm^−1^, corresponding to -CH_2_ group and the symmetric stretch of C–O-C, respectively (Supplementary Figure 2a).

### Solution thermodynamic studies

The solution thermodynamic data of 5LIO-1-Cm-3,2-HOPO was first measured to evaluate its complexation behavior with U(VI). The protonation constants of the free ligand, 5LIO-1-Cm-3,2-HOPO (denoted as LH_2_ in this part), were determined by potentiometric titrations. The formation constants could be calculated with equation (1) in the experimental section (Table 1). The two protonation constants, p*K*_a1_ and p*K*_a2,_ were measured to be 8.3(5) and 9.3(4), respectively.

**Table 1:** Protonation Constants of Ligands and Uranyl Chelation Stability Constants

Protonation Constants	EDTA(L^e^H_4_)	L^c^H_2_^a^	LH_2_	
p*K*_a1_	2.1(3)	5.91	8.3(5)	
p*K*_a2_	2.7(1)	7.14	9.3(4)	
p*K*_a3_	6.1(1)			
p*K*_a4_	9.8(1)			
Ligand	Species	m, l, h	Log*β*_mlh_	pUO_2_^b^
5LIO-1-Cm-3,2-HOPO(L)	UO_2_LH^+^	1, 1, 1	24.8(7)	16.6(5)
	UO_2_L	1, 1, 0	18.6(7)	
	UO_2_L(OH)^−^	1, 1, -1	7.5(7)	
5LIO-(Me-3,2-HOPO)(L^c^)^a^	UO_2_L^c^H^+^	1, 1, 1	18.4	15.7
	UO_2_L^c^	1, 1, 0	14.9	
	UO_2_L^c^(OH)^−^	1, 1, -1	6.3	

^a^Data from ref ^29^

^b^pM = -log [M_free_]; [M] = 10^−4^ M and [L] = 10^−3^ M

5LIO-(Me-3,2-HOPO) denoted L^c^, EDTA denoted L^e^.

The formation constants of UO_2_-L were measured by competition titration with EDTA^28^. Uranyl hydrolysis was taken into consideration for the refinement of the cumulative constants of the UO_2_-L complexes, log*β*_mlh_ calculated by equation (2). The stability constants of the UO_2_-L complexes, log*β*_mlh_, were established as log*β*_111_ = 24.8(7), log*β*_110_ = 18.6(7), and log*β*_11–1_ = 7.5(7) (Table 1). These formation constants are noticeably higher than those of the previously reported and most optimal ligand, 5LIO-(Me-3,2-HOPO) (denoted as L^c^H_2_)^29^. Only one mixed TAM-HOPO ligand has been reported with higher stability constants (log*β*_111_ = 19.75 and log*β*_11–1_ = 11.92), probably owing to the high affinity of TAM moiety for uranyl^30^. Additionally, the formation constants of 5LIO-1-Cm-3,2-HOPO complexes containing biological trace elements were also measured, including Zn–L (log*β*_111_ = 15.9(6), log*β*_110_ = 9.8(7)), Cu–L (log*β*_111_ = 16.6(5), log*β*_110_ = 9.7(4)), Ca–L (log*β*_111_ = 13.9(3), log*β*_110_ = 5.7(2)), and Mg–L (log*β*_110_ = 4.6(4)) (Supplementary Table 1). These values are significantly lower than the formation constant of UO_2_-L, indicating that 5LIO-1-Cm-3,2-HOPO is a highly selective sequestration ligand for uranyl. Furthermore, the species distribution of the M–L system based on their formation constants was calculated with *Hyss* at the defined condition of 10^−3^ M (L) and 10^−4^ M (metal ion) from pH 3.0 to 11.0 (Supplementary Figure 3). Taking the formation of uranyl-hydroxide and uranyl-carbonate complexes into consideration, the speciation diagram of the M–L system illustrates that at physiological pH (7.4), UO_2_L (94.0%) and UO_2_LH^+^ (5.9%) are the only uranyl complexes in solution. In comparison, only 2.5% MgL, 56.3% CaLH^+^, and 7.6% CaL are present in solution, suggesting that 5LIO-1-Cm-3,2-HOPO barely bind to Ca^2+^ or Mg^2+^ at physiological pH (7.4). Furthermore, the low formation constants of ZnL and CuL demonstrate that it is unlikely that ZnL and CuL complexes can form in the presence of uranyl ion, despite that 94.0% ZnL, 24.0% CuLH^+^, and 75.9% CuL exist in solution at pH 7.4 in the absence of uranyl ion. The high solubility of UO_2_L and UO_2_LH^+^ suggests that they can be easily transported and rapidly excreted from the body. Moreover, the pUO_2_ value of L, which is an assessment of the ligand affinity, was found to be 16.6(5). This value is obviously larger than those of other tetradentate HOPO ligands including 5LIO-(Me-3,2-HOPO), and potentially assigns 5LIO-1-Cm-3,2-HOPO as one of the most efficient chelators for uranyl ions^29–31^.

### Characterizations of UO_2_−5LIO-1-Cm-3,2-HOPO Complex

The ^1^H NMR and ^13^C NMR spectra of the complex in DMSO-*d*_*6*_ were collected (Supplementary Figure 1f). A slight difference between the chemical shifts of the carbon atoms from UO_2_−5LIO-1-Cm-3,2-HOPO complex and from the ligand can be observed in the ^13^C NMR spectra collected in DMSO-*d*_*6*_. More notably, the feature at 8.97 ppm in ^1^H NMR spectrum, which can be observed for the raw ligand and assigned to the hydroxyl group, disappears for the UO_2_-5LIO-1-Cm-3,2-HOPO complex, initially suggesting the complexation between the ligand and U(VI). In comparison with the FTIR spectrum of 5LIO-1-Cm-3,2-HOPO, the spectrum of UO_2_-5LIO-1-Cm-3,2-HOPO exhibits an additional peak at 899 cm^−1^ attributed to the uranyl group (Supplementary Figure 2b). A significant intensity reduction of the peak at 3282 cm^−1^ assigned to the hydroxyl group was observed for the spectrum of UO_2_−5LIO-1-Cm-3,2-HOPO, when compared with that of 5LIO-1-Cm-3,2-HOPO. Nevertheless, much more powerful evidence comes from the elemental analysis and LC-MS analysis, confirming the formation of UO_2_-5LIO-1-Cm-3,2-HOPO complex with a metal to ligand molar ratio of 1:1 (Supplementary Figure 1f).

### Extended X-ray adsorption fine structure (EXAFS)

To characterize the local coordination environment of the uranyl ion in these complexes, a solid sample of UO_2_-5LIO-1-Cm-3,2-HOPO was precipitated from a mixture of methanol and water, and was analyzed using synchrotron radiation EXAFS technique. The EXAFS spectra for the solid samples of UO_2_(NO_3_)_2_ and UO_2_-5LIO-1-Cm-3,2-HOPO contain two distinct oxygen coordination shells: axial oxygen, O_ax_, and equatorial oxygen, O_eq_ (Supplementary Figure 4). The refinement results are provided in Supplementary Table 2, listing all structural parameters, such as coordination number (CN), bonds distance (R), and the Debye/Waller factor (σ^2^). Within the experimental error, the coordination environment of uranium contains 2.0–2.2 O_ax_ atoms at bond distances ranging from 1.77–1.82 Å, and 4.70 ± 0.60 O_eq_ atoms at distances ranging from 2.41–2.48 Å. Notably, the average U–O_eq_ bond distance in UO_2_-5LIO-1-Cm-3,2-HOPO (2.41 Å) is shorter than that of UO_2_(NO_3_)_2_ (2.48 Å), whereas the U–O_ax_ bond distance in UO_2_-5LIO-1-Cm-3,2-HOPO (1.82 Å) is longer than that of UO_2_(NO_3_)_2_ (1.77 Å). These come as a result of the change in coordination numbers between UO_2_-5LIO-1-Cm-3,2-HOPO (4.7 ± 0.6) and UO_2_(NO_3_)_2_ (5.6 ± 0.6). The calculated bond distances of UO_2_-5LIO-1-Cm-3,2-HOPO are in good agreement with the structural data reported of the uranyl hydroxypyridinone compounds^25,32,33^.

### Density functional theory (DFT)

**Fig. 2:**
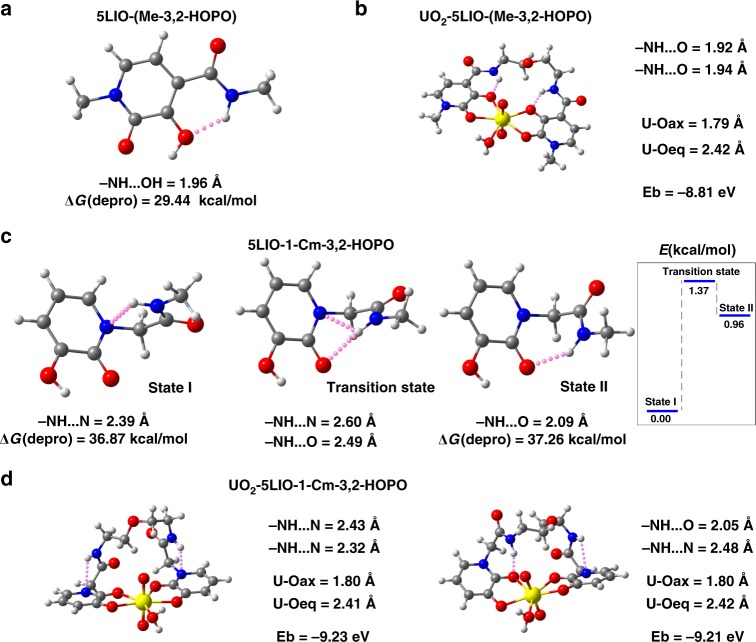
DFT optimized structures, geometric parameters, and relevant energy information. **a** 5LIO-(Me-3,2-HOPO). **b** The UO_2_-5LIO-(Me-3,2-HOPO) chelate. **c** State I and State II show two types of stable 5LIO-1-Cm-3,2-HOPO states containing –NH···N (pyridine) and –NH···O (pyridinone) intramolecular hydrogen bonds, respectively. Transition state represents the transition state between State I and State II. The inset energy diagram shows the energy relationship for all states. **d** Two types of UO_2_-5LIO-1-Cm-3,2-HOPO chelates. The left structure contains two –NH···N (pyridine) intramolecular hydrogen bonds, whereas the right contains one –NH···O (pyridinone) intramolecular hydrogen bond and one pyridine –NH···N intramolecular hydrogen bond. The gray, white, red, blue, and yellow spheres represent C, H, O, N, and U atoms, respectively. The pink dotted lines represent hydrogen bonds. ΔG(depro) in (**a**, **c**) denotes the Gibbs free energy changes of the deprotonated reactions. E_b_ in (**b**, **d**) represents the binding energies between the UO_2_^2+^ cations and the chelating ligands. The U–O_eq_ distances in b and d were calculated by averaging the four U–O distances between uranium and oxygen atoms from the chelating agents. Source Data are provided as Supplementary Data 1–3

We performed DFT calculations to reveal the effect of intramolecular hydrogen bonds on the interaction between the ligand and U(VI). In 5LIO-(Me-3,2-HOPO), the strong intramolecular hydrogen bond (approximately 1.96 Å) between the amide group and the hydroxyl group of the pyridinone ring promotes the formation of a planar local structure between the two bonding components (Fig. 2a). This strong hydrogen bond and local planar structure remain intact when chelating a UO_2_^2+^ cation (Fig. 2b). On the other hand, for 5LIO-1-Cm-3,2-HOPO, we obtained two different stable structures, one containing–NH···N (pyridine) intramolecular hydrogen bonds and the other containing –NH···O (pyridinone) intramolecular hydrogen bonds [Fig. 2c State I and State II]. Stable structure I is more energetically favorable than II by a relatively small energy difference of approximately 0.96 kcal mol^−1^. The calculated transition state (Fig. 2c) between the two structures shows a 1.37 kcal mol^−1^ energy barrier for this structural transformation. Such a low energy barrier and small energy difference imply that the C–N and C–C bonds on either side of the methylene group can freely rotate and that the two types of intramolecular hydrogen bonds in 5LIO-1-Cm-3,2-HOPO can spontaneously transform at room temperature. Compared to 5LIO-(Me-3,2-HOPO), the increase in the local degrees of freedom of 5LIO-1-Cm-3,2-HOPO can be attributed to the cooperation of the inversion of the pyridinone ring and the addition of the methylene group, leading to the weakening of the intramolecular hydrogen bonds (Fig. 2c). We then calculated the Gibbs free energy change for the deprotonation reactions (ΔG_depro_) of the hydroxyl group in the pyridinone ring within both ligands. The ΔG_depro_ values of 5LIO-1-Cm-3,2-HOPO [36.87 and 37.26 kcal mol^−1^ for I and II, respectively] are both larger than that of 5LIO-(Me-3,2-HOPO) (29.44 kcal mol^−1^), offering a qualitative explanation for the relatively larger p*K*_a_ value for 5LIO-1-Cm-3,2-HOPO determined experimentally.

Fig. 2d displays two stable UO_2_-5LIO-1-Cm-3,2-HOPO chelates, where one contains two –NH···N (pyridine) hydrogen bonds (left) while the other contains one –NH···O (pyridinone) and one –NH···N (pyridine) hydrogen bond (right). The calculated geometric parameters of both complexes (U–O_ax_ = 1.80 Å, U–O_eq_ = 2.41 Å and U–O_ax_ = 1.80 Å, U–O_eq_ = 2.42 Å) are consistent with the EXAFS measurements (U–O_ax_ = 1.82 Å, U–O_eq_ = 2.41 Å; see Supplementary Table 2). The small difference (~0.02 eV) in the values of their binding energies between the UO_2_^2+^ cation and the chelator indicates that alteration of the hydrogen-bonding scheme would not drastically affect the complexing ability of the ligand. The intramolecular hydrogen bonds transformation and minor change of the chain folding form are not expected to significantly affect the binding energy (−9.23/−9.21 eV). In contrast, the binding energy difference between UO_2_-5LIO-(Me-3,2-HOPO) and UO_2_-5LIO-1-Cm-3,2-HOPO (−8.81 eV vs −9.23/−9.21 eV) is obvious. Therefore, this calculation result reveals the enhancement of the binding ability of the 5LIO-1-Cm-3,2-HOPO ligand, especially the oxygen denticity.

**Fig. 3:**
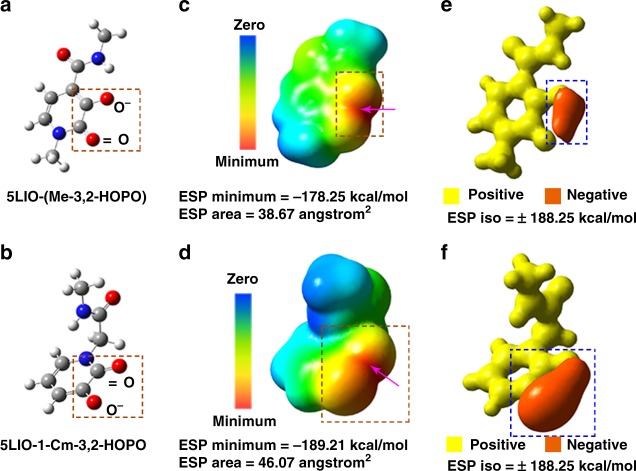
The structural and electrostatic features of 5LIO-(Me-3,2-HOPO) and 5LIO-1-Cm-3,2-HOPO. **a**, **b** Represent the deprotonated structures of 5LIO-(Me-3,2-HOPO) and 5LIO-1-Cm-3,2-HOPO, respectively. The red dotted boxes highlight the inverted carbonyl group and deprotonated hydroxyl group of the pyridinone ring. **c**, **d** Represent the ESP distributed on the electron density surface of **a**, **b** (isodensity = 0.001 a.u.). The arrows denote the global ESP minima. The red dotted boxes surround the major negative ESP areas. The negative ESP areas contributed by the two oxygen atoms were calculated and are shown below (**c**, **d). e**, **f** Represent the ESP isosurfaces at +/− 188.25 kcal mol^−1^. The blue dotted boxes surround the major spatial distribution regions at the specific ESP isosurface

We propose that the enhanced uranyl binding in 5LIO-1-Cm-3,2-HOPO originates from its intrinsic structural and electronic features. From a classical point of view, the negatively charged oxygen of the deprotonated hydroxyl group is strongly attracted to the hydrogen atom of the nearby amide in 5LIO-(Me-3,2-HOPO), thereby generating a strong –NH···O (pyridine) hydrogen bond (Fig. 3a). In contrast, when the pyridinone ring is reversed, the negatively charged oxygen of the deprotonated hydroxyl group in 5LIO-1-Cm-3,2-HOPO can be completely exposed to the environment without any intramolecular interactions (Fig. 3b). This intrinsic structural feature is expected to endow a spatial advantage in coordinating the positively charged UO_2_^2+^ cation. From a quantum perspective, DFT calculations show that, for both ligands, the global electrostatic potential (ESP) minima over the electron density surface are located between the two oxygen atoms of the pyridinone ring (Fig. 3c, d). Consequently, these two oxygen atoms act as the targeted chelating sites during complexation reactions. The calculated ESP minimum of 5LIO-1-Cm-3,2-HOPO (−189.21 kcal mol^−1^) is lower than that of 5LIO-(Me-3,2-HOPO) (−178.25 kcal mol^−1^), indicating that 5LIO-1-Cm-3,2-HOPO could provide more effective long-range electrostatic attractions for UO_2_^2+^. Moreover, the negative ESP area originating from the two oxygen atoms is further broadened over the electron density surface in 5LIO-1-Cm-3,2-HOPO (46.07 Å^2^) than in 5LIO-(Me-3,2-HOPO) (38.67 Å^2^). This observation indicates that 5LIO-1-Cm-3,2-HOPO could provide a wider effective landing surface region for UO_2_^2+^ complexation. These calculation results indicate that the exposure of the deprotonated hydroxyl group and the weak and transformable intramolecular hydrogen bond synergistically contributes to the unique distribution of the negative ESP between the two oxygen atoms. To help directly visualize and compare the ESP features, we depicted the two ESP isosurfaces for the two ligands, at a same value of +/− 188.25 kcal mol^−1^, in Fig. 3e, f. Clearly, the negative ESP distribution space is much larger in 5LIO-1-Cm-3,2-HOPO than in 5LIO-(Me-3,2-HOPO). In addition, Morokuma scheme energy decomposition analyses (EDA) results show that the E(elstat) term in UO_2_-5LIO-1-Cm-3,2-HOPO is *ca*. 1.16 eV larger than that in UO_2_-5LIO-(Me-3,2-HOPO). This result confirms our perspective that the electrostatic effect of 5LIO-1-Cm-3,2-HOPO can be fully released.

**Fig. 4:**
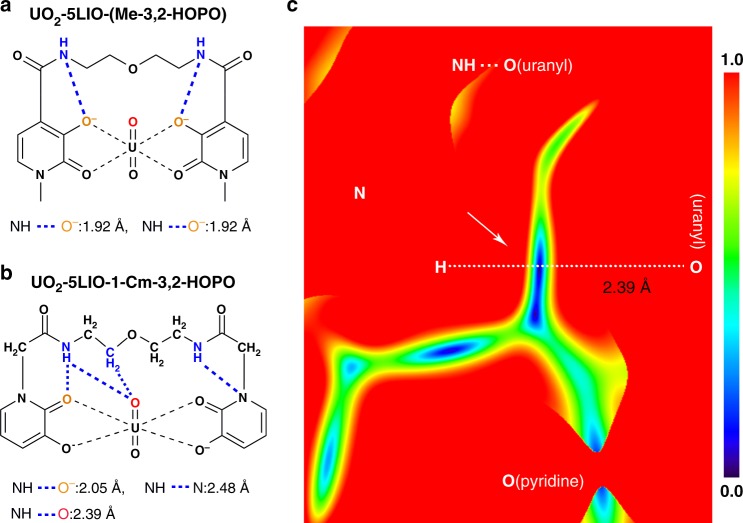
The intramolecular hydrogen bonds and RDG analysis between 5LIO-(Me-3,2-HOPO)/ 5LIO-1-Cm-3,2-HOPO and U(VI). **a** Illustration of the intramolecular –NH···O (pyridinone) hydrogen bonds in the UO_2_-5LIO-(Me-3,2-HOPO) complex. **b** Illustration of the intramolecular –NH···N (pyridine) and –NH···O (pyridinone) hydrogen bonds and intermolecular –NH···O (uranyl) hydrogen bonds in the UO_2_-5LIO-1-Cm-3,2-HOPO complex; **c** the RDG color-filled maps of the N–H–O(uranyl) and sections of **b**, respectively. The hydrogen bond distances in (**a**, **b**) are consistent with the structures of Fig. 3b (right) and **d**, respectively

Most surprisingly, DFT calculations further reveal an important interaction between the axial oxygen of uranyl and the side chain of 5LIO-1-Cm-3,2-HOPO in the UO_2_-5LIO-1-Cm-3,2-HOPO complex. For 5LIO-1-Cm-3,2-HOPO, the amide group could simultaneously form an intramolecular hydrogen bond with the oxygen of pyridinone ring, as well as an intermolecular hydrogen bond with the axial oxygen of the uranyl ion (Fig. 4a, b). The bond distance of the intramolecular hydrogen bond (–NH···O (pyridinone), 2.05 Å) is shorter than the intermolecular hydrogen bond (–NH···O (uranyl), 2.39 Å), showing relatively stronger intramolecular hydrogen bonding interactions in the former. This is mainly because of the electrostatic advantage of the oxygen of the pyridinone ring (Fig. 3d). Meanwhile, an intermolecular hydrogen bond between the methylene and the axial oxygen of uranyl can be found in the UO_2_-5LIO-1-Cm-3,2-HOPO complex. The longer –CH···O (uranyl) hydrogen bond distance (2.71 Å) reveals that this hydrogen bonding interaction is relatively weaker. Furthermore, we performed reduced density gradient (RDG) analyses based on the ground state electron density of the UO_2_-5LIO-1-Cm-3,2-HOPO complex. The small RDG values (blue areas, arrows point) between H and O (uranyl) clearly confirmed the two types of hydrogen bonding interactions, as shown in Fig. 4c. Formation of the two intermolecular hydrogen bonds are mainly attributed to the longer and more flexible side chain of the 5LIO-1-Cm-3,2-HOPO chelator. In contrast, for 5LIO-(Me-3,2-HOPO), the side chain of this chelator is relative shorter and more rigid, thus only intramolecular –NH···O (pyridine) hydrogen bonds can be formed (Fig. 2b).

In order to quantitatively evaluate the bonding contributions of the intermolecular hydrogen bond, we also performed topology analyses for the UO_2_-5LIO-1-Cm-3,2-HOPO complex (Fig. 2d, right). The UO_2_-5LIO-(Me-3,2-HOPO) complex (Fig. 2b) was also subjected to this analysis for comparison. Based on the quantum theory of atoms in molecules (QTAIM), bond critical points (BCP) between relevant H and O atoms were found in the ground state electron densities, and the potential energy density, *V*(*r*), of these BCPs were calculated. According to the relationship between *V*(*r*) and the hydrogen bond energy, *E*^HB^^34^, the *E*^HB^ can be estimated as *E*^HB^ = *V*(*r*_BCP_) / 2. It can be seen that the *E*^*HB*^ value of the intramolecular –NH···O (pyridinone) hydrogen bond in UO_2_-5LIO-(Me-3,2-HOPO) is about -0.36 eV, signifying a relatively stronger hydrogen bond interaction. This intramolecular –NH···O (pyridinone) hydrogen bond was significantly weakened to −0.27 eV in UO_2_-5LIO-1-Cm-3,2-HOPO. The *E*^HB^ values of the intermolecular –NH···O (uranyl) hydrogen bonds is −0.11 eV, implying relatively weaker intermolecular hydrogen bond interactions (Supplementary Table 3). Although the binding contributions (−0.16 eV in total) of the intermolecular hydrogen bond is relatively small compared to the total binding energy (−9.21 eV), it can be expected that the intermolecular hydrogen bond could provide an additional driving force for the coordination of uranyl and therefore enhance the chelating ability of 5LIO-1-Cm-3,2-HOPO.

### Cytotoxicity of chelating agents

**Fig. 5:**
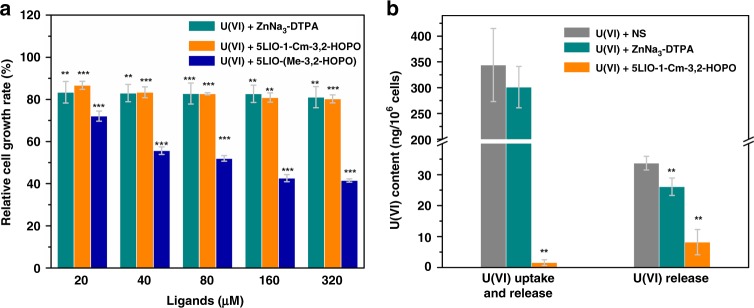
The comprehensive cytotoxicity and uranium removal efficiency of NRK-52E cells. **a** Dosage-dependent cell growth rate of NRK-52E cells treated with U[(VI), 12.4 μM] + 5LIO-1-Cm-3,2-HOPO, U[(VI), 12.4 μM] + 5LIO-(Me-3,2-HOPO), and U[(VI), 12.4 μM] + ZnNa_3_-DTPA, respectively. *p < 0.05, **p < 0.01, ***p < 0.001 vs control, paired-sample *T* test for independent-samples, *n* = 6 samples. Bars indicate SD, *n* = 6 samples. **b** Effects of ligand on U(VI) uptake and release when ligand and U(VI) are given together, and on U(VI) release in NRK-52E cells when U(VI) and ligand are given one after the other. Source data are provided as a Source Data file. *p < 0.05, **p < 0.01, ***p < 0.001 vs U(VI)-treated control, paired-sample *T* test for independent-samples, *n* = 3 samples. Bars indicate SD, *n* = 3 samples

Given the high affinity of 5LIO-1-Cm-3,2-HOPO for uranyl, the U(VI) sequestration performance and toxicity of 5LIO-1-Cm-3,2-HOPO were evaluated and compared with those of the clinically-used ZnNa_3_-DTPA and the previously reported most optimal tetradentate ligand 5LIO-(Me-3,2-HOPO) in vitro and in vivo. Renal injury is one of the major concerns in the case of uranium contamination. Therefore, U(VI) uptake and release assays were first conducted using renal proximal tubular epithelial cells from rat (NRK-52E cells). The toxicity assay of U(VI) with chelation therapy agent has been performed, and 12.4 μM was considered as the acceptable concentration for the following cellular assays^35^. Then, a comprehensive toxicity assay of U(VI) and chelating agents was performed by adding 12.4 μM U(VI) and different concentrations of chelating agents ranging from 20.0 to 320.0 μM. The results show that the comprehensive toxicity of UO_2_-5LIO-1-Cm-3,2-HOPO is slightly lower than that of UO_2_-ZnNa_3_-DTPA at low dosage, and notably lower than that of UO_2_-5LIO-(Me-3,2-HOPO) (Supplementary Table 4, Fig. 5a).

### U(VI) uptake and release

In vitro U(VI) uptake and release assays were conducted to investigate the uranyl removal efficiency of 5LIO-1-Cm-3,2-HOPO at the cellular level. In vitro assay for U(VI) uptake and release from NRK-52E cells were performed by adding 12.4 μM U(VI) solution to cells, followed by 320.0 μM solution of the chelators. Fig. 5b shows that the addition of 5LIO-1-Cm-3,2-HOPO can remove 99.5% of uranium from NRK-52E cells, whereas ZnNa_3_-DTPA can remove only 12.5% of uranium under the same condition. Clearly, the uranium removal efficiency of 5LIO-1-Cm-3,2-HOPO is much higher than that of ZnNa_3_-DTPA at an equal molar dosage (Supplementary Table 5). However, since the NRK-52E cells were cultured in medium that contain uranium throughout the experiment, question remains as whether the ligand mainly play the role of preventing the uranium uptake of the cells, or enhancing the release of the intracellular uranium. Therefore, another in vitro assay for U(VI) release from NRK-52E cells was designed by adding 12.4 μM U(VI) solution to cells first, then removing the U(VI) culture medium, followed by the addition of 320.0 μM solution containing the chelators. As shown in Fig. 5b, the treatment of 5LIO-1-Cm-3,2-HOPO resulted in a uranium removal efficiency of 75.8%, illustrating that 5LIO-1-Cm-3,2-HOPO plays a major role for enhancing the U(VI) release from cells (Supplementary Table 6).

### In vivo uranyl decorporation

**Fig. 6:**
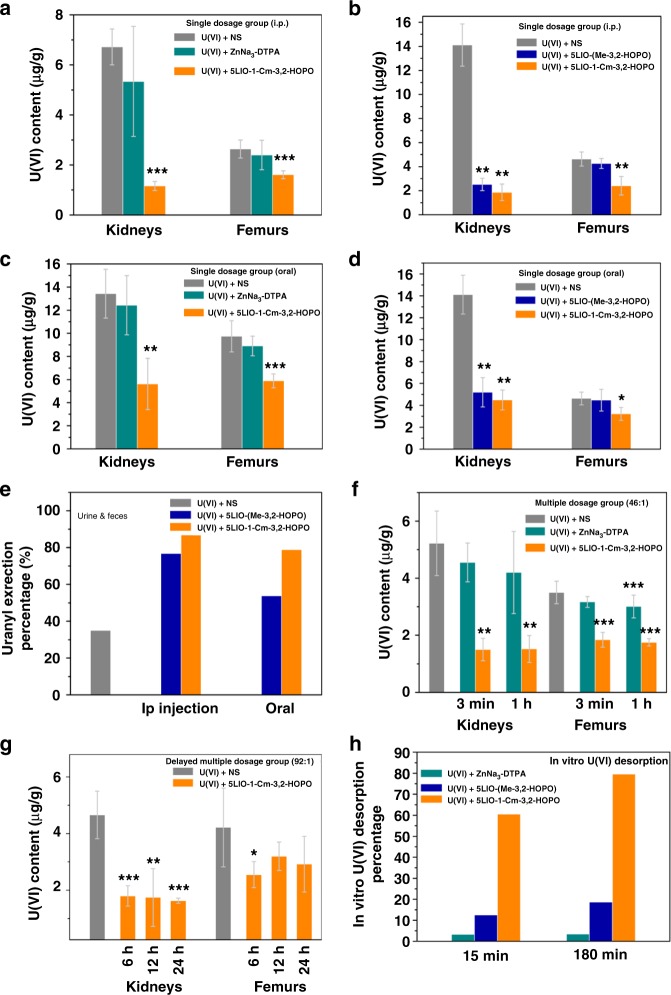
In vivo decorporation efficiency and in vitro desorption efficiency results. **a**–**d** Removal efficiency of U(VI) deposited in the kidneys and femurs compared to the control group. For the single-dose group, mice were ip injected (193 μmol kg^−1^, molar ratio 92:1) or oral administered (644 μmol kg^−1^, molar ratio 307:1) with ligands after the iv injection of U(VI) (0.5 mg U(VI) kg^−1^) and then were killed 24 h later. **e** The urine & feces excretion percentage for the group 5LIO-1-Cm-3,2-HOPO and 5LIO-(Me-3,2-HOPO) treated with single-dose ip injection or oral administration. **f** Removal efficiency of U(VI) deposited in the kidneys and femurs compared to the control group, for the multiple-dose group. Mice were given the ligand by ip (97 μmol kg^−1^, molar ratio 46:1) injection at 3 min, 6 h, 24 h, and 48 h, or at 1 h, 7 h, 25 h, and 49 h after the iv injection of U(VI) (0.5 mg U(VI) kg^−1^) and were killed 72 h later. **g** Removal efficiency of U(VI) deposited in the kidneys and femurs compared to the control group, for the delayed multiple-dose group. Mice were given the ligand by ip (193 μmol kg^−1^, molar ratio 92:1) injection at 6 h, 12 h, 30 h, and 54 h (6 h delayed multiple-dose group), or at 12, 18, 36, and 60 h (12 h delayed multiple-dose group), or at 24, 30, 48, and 72 h (24 h delayed multiple-dose group) after the iv injection of U(VI) (0.5 mg U(VI) kg^−1^) and were killed 7 d later. *p < 0.05, **p < 0.01, ***p < 0.001 vs. U(VI)-treated control, paired-sample T-test for independent-samples, *n* = 5 mice. Bars indicate SD, *n* = 5 mice. **h** U(VI) desorption efficiency from HAP using 5LIO-1-Cm-3,2-HOPO, 5LIO-(Me-3,2-HOPO) or ZnNa_3_-DTPA. Source data are provided as a Source Data file

Considering the remarkable performance of 5LIO-1-Cm-3,2-HOPO in removing U(VI) at the cellular level, further evaluation of 5LIO-1-Cm-3,2-HOPO, 5LIO-(Me-3,2-HOPO), and ZnNa_3_-DTPA was conducted via in vivo chelation of U(VI) in mice. Fig. 6 illustrates the procedure of rounded in vivo decorporation assays with different administration methods and time. Three batches of in vivo decorporation assays were designed to study and compare the performance between the three ligands, 5LIO-1-Cm-3,2-HOPO, 5LIO-(Me-3,2-HOPO), and ZnNa_3_-DTPA, including single dosage group with intraperitoneal (ip) injection, single dosage group with oral administration, and multiple dosage and delayed multiple dosage groups with ip injection.

In the single-dose group with intraperitoneal (ip) injection, the first assay was performed to compare the performance of 5LIO-1-Cm-3,2-HOPO and ZnNa_3_-DTPA on the decorporation of uranium, where 5LIO-1-Cm-3,2-HOPO and ZnNa_3_-DTPA (193 μmol kg^−1^, molar ratio to uranium is 92:1) were injected intraperitoneally (ip) three min after an initial intravenous (iv) injection of U(VI) (0.5 mg kg^−1^). At 24 h after this iv injection, the kidneys and femurs from the control group were determined to contain 6.72 ± 0.72 and 2.64 ± 0.36 μg U(VI) per gram of tissue, respectively. The group receiving the ip injection of 5LIO-1-Cm-3,2-HOPO displayed a reduced accumulation of U(VI) in the kidneys and femurs, where uranium levels were decreased by 82.9 and 39.0%, respectively. The groups given ZnNa_3_-DTPA, however, showed limited removal of U(VI) under identical experimental conditions (20.5% and 9.1%, respectively) (Fig. 6a and Supplementary Table 7). Another assay was performed to directly compare the performance of 5LIO-1-Cm-3,2-HOPO and 5LIO-(Me-3,2-HOPO) (193 μmol kg^−1^, molar ratio to uranium is 92:1) on decorporation of uranium in a similar fashion. The group treated with 5LIO-1-Cm-3,2-HOPO yielded a 86.8% and 47.9% of U(VI) removal efficiency in the kidneys and femurs, respectively. Notably, this ligand shows a removal efficiency in the femurs six times higher than that of 5LIO-(Me-3,2-HOPO), since the group given 5LIO-(Me-3,2-HOPO) showed limited effect on removing U(VI) deposited in femurs under the identical experimental conditions (Removal percentage of 8.0%) (Supplementary Table 8 and Fig. 6b). These results are well consistent with the previous study when 5LIO-(Me-3,2-HOPO) was originally synthesized^15,23^. The results of uranium concentrations in urine and feces for the groups treated with 5LIO-1-Cm-3,2-HOPO and 5LIO-(Me-3,2-HOPO) via ip injection are listed and compared in Supplementary Table 9, showing a clear increase of uranium excretion in the 5LIO-1-Cm-3,2-HOPO treated group. This further supports the observation of the enhanced decorporation efficiency of 5LIO-1-Cm-3,2-HOPO, in comparison with 5LIO-(Me-3,2-HOPO). The other in vivo assays were performed to compare the decorporation ability between 5LIO-1-Cm-3,2-HOPO, 1-Hydroxyethylidene-1,1-diphosphonic acid (HEDP), and NaHCO_3_, which have been reported to be effective for U(VI) decorporation^36,37^. Supplementary Table 10 lists the U(VI) removal performance in kidneys and femurs following the same experimental procedure. The group treated with HEDP showed reduction of 31.2% of U(VI) deposited in kidneys and 17.4% of U(VI) deposited in femurs, respectively. The group treated with NaHCO_3_ showed very limited effect on removing U(VI) from mice.

More importantly, in vivo assay with single-dose oral administration was performed to evaluate the oral efficiency of the 5LIO-1-Cm-3,2-HOPO, 5LIO-(Me-3,2-HOPO), and ZnNa_3_-DTPA. Similiarly, an assay with single-dose oral administration was first performed to compare the performance of 5LIO-1-Cm-3,2-HOPO and ZnNa_3_-DTPA. 5LIO-1-Cm-3,2-HOPO and ZnNa_3_-DTPA (644 μmol kg^−1^, molar ratio to uranium is 307:1) were orally administered by gastric tube three min after an initial intravenous (iv) injection of U(VI) (0.5 mg kg^−1^). The group received the oral administration of 5LIO-1-Cm-3,2-HOPO showed a reduction of 58.2% uranium from kidneys and 39.5% from femurs (Supplementary Table 11 and Fig. 6c). Subsequently, a comparison between 5LIO-1-Cm-3,2-HOPO and 5LIO-(Me-3,2-HOPO) was conducted in a similar fashion. As shown in Fig. 6d, the group given 5LIO-1-Cm-3,2-HOPO displayed similar U(VI) removal percentage in kidneys (68.2%) with the group given 5LIO-(Me-3,2-HOPO) (63.2%), but a much higher U(VI) removal ratio in femurs (30.5%) was observed for the group of 5LIO-1-Cm-3,2-HOPO than the group of 5LIO-(Me-3,2-HOPO) (3.5%) (Supplementary Table 12). Fig. 6e shows the excretion results of uranium concentrations in urine and feces for the groups orally treated with 5LIO-1-Cm-3,2-HOPO and 5LIO-(Me-3,2-HOPO). For the mice given ip injection, 76.8% and 86.9% of uranium were excreted in the group treated with 5LIO-(Me-3,2-HOPO) and 5LIO-1-Cm-3,2-HOPO, respectively, which correspond to enhancement of 2.2- and 2.5-fold comparing to that of the control group (35.1%). For the oral administration group, 53.8% and 78.9% of uranium was excreted in the group treated with 5LIO-(Me-3,2-HOPO) and 5LIO-1-Cm-3,2-HOPO, respectively, corresponding to an enhanced excretion of 1.5- and 2.3-fold in comparison to the excretion of the control group, respectively. In comparison with the ip injection groups, U(VI) excretion efficiency is lower in the oral administration groups of both ligands, consistent with the relatively lower U(VI) decorporation efficiency in kidneys and femurs in the orally treated groups (Supplementary Table 12).

In the multiple-dose group, 5LIO-1-Cm-3,2-HOPO and ZnNa_3_-DTPA (97 μmol kg^−1^; molar ratio to uranium of 46:1) were ip injected at 3 min, 6 h, 24 h, and 48 h after the initial iv injection of U(VI). At 72 h after the iv injection of 0.5 mg U(VI) kg^−1^, the kidneys and femurs from the control group contained 5.22 ± 1.13 and 3.50 ± 0.39 μg U(VI) per gram of tissue, respectively (Fig. 6f). Compared with the control group, 5LIO-1-Cm-3,2-HOPO significantly reduced U(VI) levels in the femur by 47.4%. Another multiple-dose assay was conducted by ip injection of 5LIO-1-Cm-3,2-HOPO and ZnNa_3_-DTPA (97 μmol kg^−1^; molar ratio to uranium is 46:1) at 1, 7, 25, and 49 h after the initial iv injection of U(VI). Similarly, kidneys and femur samples were obtained 72 h after the initial U(VI) injection. Notably, 70.9% of U(VI) deposited in the kidneys and 50.0% in femurs were removed in the 5LIO-1-Cm-3,2-HOPO group compared with the untreated U(VI)-injected controls (Fig. 6f). These values are almost identical with those of the multiple-dose group treated 3 min after the U(VI) injection. This achievement overcomes a large hurdle in developing actinide chelation agents and opens up the possibility of complete uranium removal from bones by increasing the dosage further (Supplementary Table 13).

In most nuclear accidents, an immediate response to radionuclide introduction into human bodies is not applicable. Therefore, decorporation agents that can still function despite delayed administration are preferred for chelation therapy with elevated significance. To fully study the relationship between the decorporation efficiency and the time of administration, 5LIO-1-Cm-3,2-HOPO (193 μmol kg^−1^; molar ratio to uranium is 92:1) was given via ip injection at the following three different time intervals: (1) 6, 12, 30, and 54 h; (2) 12, 18, 36, and 60 h; (3) 24, 30, 48, and 72 h, after the initial iv injection of U(VI). The three groups with 6, 12, and 24 h delayed multiple-dose administration display a similar removal level of U(VI) from kidneys of 61.4%, 62.4%, and 65.0%, respectively. For the decorporation efficiency in femurs, all three groups significantly reduced U(VI) accumulation in femurs by 39.6, 24.2, and 30.8%, respectively (Fig. 6g and Supplementary Table 14), suggesting that the decorporation efficiency is nearly independent of the time of administered treatment.

Hydroxyapatite, or HAP, is the main inorganic mineral component in bones and can form highly stable and insoluble complexes with the uranyl cation. Therefore, an in vitro desorption experiment was designed to demonstrate the thermodynamic possibility of 5LIO-1-Cm-3,2-HOPO desorbing the complexed uranyl from HAP to further support the experimental performance of the ligand removing uranium from bones. For comparison, the desorption experiments using 5LIO-(Me-3,2-HOPO) and ZnNa_3_-DTPA were also conducted using the same method. As shown in Fig. 6h, after that the sorption equilibrium between U(VI) and HAP had been reached after 180 min, the addition of 5LIO-1-Cm-3,2-HOPO resulted in rapid uranyl desorption from HAP with a high desorption rate of 79.6%. This signifies that the process of decorporating uranium from bone with 5LIO-1-Cm-3,2-HOPO is indeed thermodynamically favorable. In sharp contrast, 5LIO-(Me-3,2-HOPO) and ZnNa_3_-DTPA only resulted in a desorption rate of 18.6 and 3.4%, respectively.

## Discussion

The results described above demonstrate that weakening the intramolecular hydrogen bonds between the oxygen donor sites within the HOPO ligand can significantly increase their uranium coordination capabilities. According to this simple idea, we have successfully synthesized a unique chelating ligand, 5LIO-1-Cm-3,2-HOPO. The combination of solution titration studies, EXAFS analysis, FTIR analysis, NMR analysis, LC-MS analysis, elemental analysis, and first principles theoretical analysis demonstrates the coordination mode and the thermodynamic feasibility for decorporating uranium from kidneys and bones. DFT calculations indicate that with the additional acyl derivative on the ring nitrogen, the formation of strong intramolecular hydrogen bond is greatly prohibited, giving rise to strengthened uranium-HOPO interaction consequently. In vitro U(VI) removal assays results show that 5LIO-1-Cm-3,2-HOPO can significantly enhance the release of intracellular U(VI) release from cells. Further, in vivo uranium decorporation assays demonstrate that this ligand not only improve the reduction of U(VI) levels in kidneys, but also show a record high removal efficiency of uranium from bones even for the oral and delayed treatments, while maintaining a low toxicity at the similar level with ZnNa_3_-DTPA. Overall, these results signify that 5LIO-1-Cm-3,2-HOPO represents one of the most promising U(VI) decorporation agents and its practical application is expected to be realized in the near future.

## Methods

### Reagents and materials

Caution! ^238^UO_2_(NO_3_)_2_·6H_2_O used in this study is radioactive and standard procedures for handling radioactive materials were performed throughout these experiments. 1,2-dihydro-2,3-pyridinediol (98%, TCI), 1,5-diamino-3-oxapentane (99%, TCI), benzyl chloride (98%, J & K), ethyl bromoacetate (99%, Adamas), N-hydroxysuccinimide (NHS), 1-(3-dimethylaminopropyl)-3-ethylcarbodiimide hydrochloride (EDC) (99%, J&K), and 5% Pd/C (Adamas) were all used as received. 5LIO-(Me-3,2-HOPO)Bn (98%) was purchased from the company of Shandong Huijing Bio-Pharmatech Co., Ltd.

### Stock solution of 5LIO-1-Cm-3,2-HOPO and uranyl

5LIO-1-Cm-3,2-HOPO or 5LIO-(Me-3,2-HOPO) (12.9 mg) was dissolved in DMSO (1.0 mL) and UO_2_(NO_3_)_2_·6H_2_O (49.9 mg) was`dissolved in 1.0 mL ultrapure water, followed by sterilization with a 0.22 μm sterilization filter. The work solutions containing chelating agent and U(VI) were prepared by diluting with culture medium to the expected concentrations for cell treatment.

### Cell line and culture

The NRK-52E cell line (organism: kidney, rat, ATCC® CRL-1571™) was purchased from Maisha Bio-Technology, Ltd. and cultured in a medium mixture of F-12 nutrient mixture (DMEM/F-12) (Hyclone, Thermo Scientific), 10% (v/v) fetal bovine serum (FBS) (Gibco, Invitrogen Technologies), and 1% Penicillin-Streptomycin in a humidified atmosphere of 5% CO_2_ at 37 °C. The cells were propagated every two days.

### Ligand synthesis

The ligand, 5LIO-1-Cm-3,2-HOPO, was obtained from a 4-step synthesis (Fig. 1).

1,2-dihydro-2,3-pyridinediol (22.6 g, 0.2 mol) and ethyl bromoacetate (133.6 g, 0.8 mol) were stirred together, then flushed with nitrogen for 1 h and refluxed under nitrogen for 24 h. The solution was allowed to cool down to room temperature, yielding a beige precipitate. The solution was filtered and the product was washed with acetone for at least three times. Recrystallization from 95% ethanol yielded colorless needle-shape crystals, Ethyl 2-(3-hydroxy-2-oxopyridin-1(2H)-yl)acetate (A) (27.4 g, 70%). ^1^H NMR (400 MHz, DMSO): δ 9.14 (s, 1H), 7.13 (d, *J* = 6.8 Hz, 1H), 6.72 (d, *J* = 7.2 Hz, 1H), 6.11 (t, *J* = 7.0 Hz, 1H), 4.71 (s, 2H), 4.14 (dd, *J* = 5.3 Hz 2H), 1.20 (t, *J* = 7.0 Hz, 3H); ^13^C NMR (100 MHz, DMSO):168.5, 158.4, 147.1, 129.3, 115.8, 105.7, 61.4, 50.8, 14.5; ATR-FTIR: 3236 cm^−1^ (*v*_-OH_), 2972 cm^−1^ (*v*_-CH_), 1741 cm^−1^ (*v*_-C = O_), 1654 cm^−1^ (*v*_-C = O_), 1204 cm^−1^ (δ_-C–O–C_), 698 cm^−1^ (δ_-C–C_); Anal. Calcd (found) for C_9_H_11_NO_4_ (197.19): C, 54.82(53.64); H, 5.62 (5.52); N, 7.10 (6.92); LC-MS [M + H^+^] *m/z*: 197.86 (Supplementary Figure 1a and Supplementary Figure 2a).

Ethyl 2-(3-hydroxy-2-oxopyridin-1(2H)-yl)acetate (A) (10.0 g, 50.0 mmol) was placed into a 500.0 mL round-bottom flask and dissolved in 300.0 mL of 90% methanol; the pH of the solution was adjusted to 12 with NaOH aqueous solution (0.1 M). Benzyl chloride (25.0 g, 0.2 mol) was added to this and the solution was refluxed for 12 h, while maintaining a pH above 12 with the addition of NaOH aqueous solution until the reaction completed. The solution was cooled down to room temperature and methanol was removed by rotary evaporation. The aqueous solution was extracted with dichloromethane (3 × 50.0 mL), diluted with 100.0 mL of H_2_O and the pH was adjusted to 1.0 with concentrated hydrochloric acid. The crude product that precipitated out was filtered. Recrystallization from 95% ethanol yielded colorless needle-shape crystals, 2-(3-benzyloxy)-2-oxopyridin-1(2H)-yl)acetic acid (B) (10.8 g, 83%). ^1^H NMR (400 MHz, DMSO): δ 7.44–7.32 (m, 5H), 7.26 (d, *J* = 6.4 Hz, 1H), 6.93 (d, *J* = 7.2 Hz, 1H), 6.15 (t, *J* = 7.2 Hz, 1H), 5.01 (s, 2H), 4.61 (s, 2H); ^13^C NMR (100 MHz, DMSO):169.8, 157.5, 148.3, 136.7, 131.2, 128.8, 128.4, 128.4, 116.1, 104.2, 70.2, 51.0; ATR-FTIR: 2905 cm^−1^ (*v*_-OH_), 2651 cm^−1^ (*v*_-CH_), 1737 cm^−1^ (*v*_-C = O_/_-COOH_), 1648 cm^−1^ (*v*_-C = O_), 1571 cm^−1^ (δ_-OH_), 749 cm^−1^ (δ_-C–C_); Anal. Calcd (found) for C_14_H_13_NO_4_(259.08): C, 64.86(64.76); H, 5.05 (5.582); N, 5.40 (5.372); LC-MS [M + H^+^] *m/z*: 259.73 (Supplementary Figure 1b and Supplementary Figure 2a).

A solution of 2-(3-benzyloxy-2-oxopyridin-1(2H)-yl)acetic acid (B) (5.0 g, 19.0 mmol) in *N*,*N*-Dimethylformamide (DMF, 100.0 mL) was stirred in an ice bath. N-hydroxysuccinimide (NHS, 2.3 g, 20.0 mmol) was dissolved in this solution, followed by the addition of 1-(3-Dimethylaminopropyl)-3-ethylcarbodiimide hydrochloride (EDC, 3.8 g, 20.0 mmol). Then the mixture was stirred for 4 h at room temperature. Then, the linker solution, 1,5-diamino-3-oxapentane (0.9 g, 8.3 mmol) in 5.0 mL DMF, was added gradually and subsequently stirred at room temperature overnight. The solvent was removed in vacuo and 50.0 mL H_2_O was added to the crude product, followed by stirring. A white crude product precipitated and was filtered. The product was dried at 50 °C under vacuum to yield 5LIO-1-Cm-3,2-HOPOBn (N,N’-[oxybis(ethane-2,1-diyl)]bis[2-(3-benzyloxy-2-oxopyridin-1(2H)-yl)acetamide]) (4.4 g, 82%). ^1^H NMR (400 MHz, DMSO): δ 8.15 (t, *J* = 4.8 Hz, 1H), 7.44–7.32 (m, 5H), 7.17 (d, *J* = 6.8 Hz, 1H), 6.89 (d, *J* = 6.8 Hz, 1H), 6.09 (t, *J* = 7.2 Hz, 1H), 4.98 (s, 2H), 4.49 (s, 2H), 3.12 (dd, *J* = 4.8 Hz, 2H), 1.65–1.61(m, 2H); ^13^C NMR (100 MHz, DMSO): 167.3, 157.1, 148.3, 137.0, 131.8, 128.8, 128.4, 128.3, 116.1, 116.0, 103.9, 103.8, 70.2, 69.1, 51.6, 39.1; ATR-FTIR: 2908 cm^−1^ (*v*_-CH_), 1633 cm^−1^ (*v*_-C = O_/δ_–NH_), 1591 cm^−1^ (δ_–NH_), 1252 cm^−1^ (δ_-C-O_), 738 cm^−1^ (δ_-C–C_); Anal. Calcd (found) for C_32_H_34_N_4_O_7_(586.24): C, 65.88(64.94); H, 6.20 (5.887); N, 9.31 (9.36); LC-MS [M + Na^+^] *m/z*: 609.02 (Supplementary Figure 1c and Supplementary Figure 2a).

5% Pd/C was added slowly to a solution of 5LIO-1-Cm-3,2-HOPOBn (4.0 g, 6.8 mmol) dissolved in 100 mL methanol. The mixture was then stirred under H_2_ at room temperature for 4 h. After filtration, the product was re-dissolved in DMF and filtered. The filtrate was subjected to rotary evaporation to give a pale gray solid. The product was dried at 50 °C under vacuum to yield the final product 5LIO-1-Cm-3,2-HOPO (N,N'-[oxybis(ethane-2,1-diyl)]bis[2-(3-hydroxy-2-oxopyridin-1(2H)-yl)acetamide]) (2.5 g, 90%). ^1^H NMR (400 MHz, DMSO): δ 8.96 (s, 1H), 8.22 (t, J = 4.8 Hz, 1H), 7.09 (d, J = 6.8 Hz, 1H), 6.70 (d, *J* = 6.8 Hz, 1H), 6.07 (t, *J* = 7.0 Hz, 1H), 4.57 (s, 2H), 3.44(t, *J* = 5.4 Hz, 2H), 3.28–3.24 (dd, *J* = 4.8 Hz, 2H); ^13^C NMR (100 MHz, DMSO): 167.1, 158.3, 147.0, 129.2, 115.3, 105.1, 67.9, 51.6, 36.3; Anal. Calcd (found) for C_18_H_22_N_4_O_7_(406.39): C, 53.2 (54.64); H, 5.46 (5.56); N, 13.79 (13.49); ATR-FTIR: 3273 cm^−1^ (*v*_-OH_), 2925 cm^−1^ (*v*_-CH_), 1644 cm^−1^ (*v*_-C = O_/δ_–NH_), 1554 cm^−1^ (δ_–NH_), 1252 cm^−1^ (δ_-C-O_); LC-MS [M+H^+^] *m/z*: 406.79 and [M+Na^+^] *m/z*: 428.78 (Supplementary Figure 1d and Supplementary Figure 2a).

This compound was prepared following the procedure of 5LIO-1-Cm-3,2-HOPO. 5LIO-(Me-3,2-HOPO)Bn (150  mg, 0.25 mmol) was dissolved in 20.0 mL MeOH with the addition of 5% Pd/C. The mixture was then stirred under H_2_ at room temperature for 4 h. The separation and purification were performed as described above. The product was dried at 50 °C under vacuum to yield the final product 5LIO-(Me-3,2-HOPO) (1,5-bis[(3-hydroxy-1-methyl-2-oxo-1,2-dihydro-pyridin-4-yl)carboxamido]) (96 mg, 93%). ^1^H NMR (400 MHz, DMSO): δ 8.50 (s, 1H), 7.17 (d, *J* = 7.2 Hz, 1H), 6.52 (d, *J* = 7.2 Hz, 1H), 3.55 (t, *J* = 5.2 Hz, 2H), 3.47 (s, 3H), 3.39(t, 2H); ^13^C NMR (100 MHz, DMSO): 165.7, 158.36, 147.8, 128.2, 117.6, 103.2, 69.0, 39.3, 37.3; ATR-FTIR: 3380 cm^−1^ (*v*_-OH_), 2915 cm^−1^ (*v*_-CH_), 1644 cm^−1^ (*v*_-C=O_/δ_–NH_), 1591 cm^−1^ (δ_–NH_), 1219 cm^−1^ (δ_-C-O_); Anal. Calcd (found) for C_18_H_22_N_4_O_7_(406.39): C, 53.2 (52.48); H, 5.46 (5.47); N, 13.79 (13.77); LC-MS [M+H^+^] *m/z*: 406.89 (Supplementary Figure 1e and Supplementary Figure 2a).

### Potentiometric Titrations

All stock solutions for potentiometric titrations were prepared using ultrapure water with a conductivity of 18.2 mΩ cm^−1^ boiled for two hours, while being purged with nitrogen overnight. 0.1 M KCl was prepared by dissolving an appropriate amount of salt into the carbonate-free water, and 0.1 M HCl and 0.1 M KOH stock solutions were obtained commercially and standardized with hydrogen phthalate. All stock solutions for titration are protected under nitrogen. The glass electrode (Metrohm Microtrode) used for the potentiometric measurements was calibrated by first adding 2.0 mL of standardized 0.1 M HCl in 25.0 mL of 0.1 M KCl, and then titrating using standardized 4.0 mL of 0.1 M KOH. The data was analyzed with the program GLEE to obtain values of the *E*° and slope, which is then used to update the electrode parameters in the Tiamo software. All the titrations were performed with the apparatus of Metrohm 905 at 25 ^o^C, and the titration instruments were fully automated and controlled using Tiamo software.

The ligand’s protonation constants was performed by dissolving 5LIO-1-Cm-3,2-HOPO (25.0 mg, 0.06 mmol) in 0.1 M KCl (contains 5% DMSO). The solution’s pH was adjusted to about 2.5 by adding 2.0 mL of 0.1 M HCl, and then titrated using 4.0 mL of 0.1 M KOH with a 0.03 mL increment. The minimum and maximum equilibration time between additions of titrant was 60 and 240 s, respectively. The ligand’s protonation constants were determined by three independent experiments. Data were analyzed by nonlinear least-squares program (hyperquad 2013) and the results are listed in Table 1. The ligand protonation constants, p*K*_as_, define by equation (1) are 8.3(5) and 9.3(4), respectively.

Formation constants for UO_2_-L were determined by competition titration experiments with EDTA. Initially for the potentiometric experiments, about 5.0 mg UO_2_ (NO_3_)_2_·6H_2_O, 4.1 mg 5LIO-1-Cm-3,2-HOPO, and 3.7 mg EDTA-Na_2_ (1:1:1, at ~10 μmol) were dissolved in 25.0 mL 0.1 M KCl. The solution’s pH was adjusted to about 2.5 by adding 2.0 mL of 0.1 M HCl, and then titrated using 4.0 mL of 0.1 M KOH with a 30.0 μL increment. The minimum and maximum equilibration time between additions of titrant was 60 and 240 s, respectively. The formation constants for UO_2_-L were determined by three independent experiments. In addition, the formation constants of biologically trace elements, (M(II) = Zn(II), Ca(II), Mg(II), and Cu(II)), with 5LIO-1-Cm-3,2-HOPO were measured to investigate the binding affinities of L with these metal elements. Similarly, 5LIO-1-Cm-3,2-HOPO and M(II) (1:1, at approx. 10 μmol) were all dissolved in 25.0 mL 0.1 M KCl (containing 5% DMSO). Potentiometric titrations were accomplished from low to high pH (2.5–11). Data were also refined by nonlinear least-squares and the results are included in Table 1 and Supplementary Table 1.

The species distribution of 5LIO-1-Cm-3,2-HOPO and metal ions were calculated with *Hyss* at the condition of 10^−3^ M L and 10^−4^ M metal ions from pH 3 to 11 (Supplementary Figure 3), taking the formation of uranyl-hydroxide and uranyl-carbonate complexes into consideration.

1 $$pK_{{\rm{an}}} = - \log \frac{{[LH_n]}}{{[H][LH_{n - 1}]}} = \log \beta _{01n} - \log \beta _{01n - 1}$$

2 $$m{\mathrm{M}}^{2 + } + l{\mathrm{L}}^{2 - } + h{\mathrm{H}}^ + = {\mathrm{M}}_m{\mathrm{L}}_l{\mathrm{H}}_h^{(2m - l + h) + }.$$

Supplementary Figure 5 shows the refinement details for the cumulative constants of 5LIO-1-Cm-3,2-HOPO and metal elements with nonlinear least-squares program (hyperquad 2013). For the UO_2_-L system, although the formation constants measurement of UO_2_-L was carried out by competition titration with EDTA (denoted L^e^H_4_), only a small quantity of UO_2_L^e^H^+^ and UO_2_L^e^ exist at low pH. The results suggest that EDTA is not able to compete with 5LIO-1-Cm-3,2-HOPO (Supplementary Figure 5a).

### The preparation of UO_2_-5LIO-1-Cm-3,2-HOPO complex

A solution of 5LIO-1-Cm-3,2-HOPO (40.6 mg, 0.1 mmol) in 0.5 mL DMSO was added to 10 mL of UO_2_(NO_3_)_2_·6H_2_O (50.2 mg, 0.1 mmol) while being stirred. The pale yellow solution immediately turned orange. These conditions support the formation of a UO_2_-5LIO-1-Cm-3,2-HOPO complex. The transparent reaction mixture became turbid after the addition of 3 or 4 drops of 6 M KOH solution. The orange UO_2_-5LIO-1-Cm-3,2-HOPO complex was collected by filtration, washed with H_2_O, and dried in a vacuum oven to yield the UO_2_-5LIO-1-Cm-3,2-HOPO complex (60 mg, 87%). ^1^H NMR (400 MHz, DMSO): δ 8.23 (t, *J* = 5.2 Hz, 1H), 7.06 (d, *J* = 6.8 Hz, 1H), 6.69 (d, *J* = 6.8 Hz, 1H), 6.07 (s, 1H), 4.57 (s, 2H), 3.44 (t, J = 5.4 Hz, 2H), 3.28–3.26 (dd, *J* = 4.8 Hz, 2H); ^13^C NMR (100 MHz, DMSO): 167.3, 158.5, 147.3, 129.7, 115.6, 105.1, 69.1, 51.7, 39.2; Anal. Calcd (found) for UO_2_(C_18_H_22_N_4_O_7_)·H_2_O (692.42): C, 31.22 (30.98); H, 3.20 (3.29); N, 8.09 (7.89); ATR-FTIR: 3282 cm^−1^ (*v*_-OH_), 2947 cm^−1^ (*v*_-CH_), 1661 cm^−1^ (*v*_-C = O_), 1286 cm^−1^ (δ_-C-O-C_), 899 cm^−1^ (δ_-U = O_); LC-MS [M-H_2_O+H^+^] *m/z*: 675.21 (Supplementary Figure 1f and Supplementary Figure 2b).

### ATR-FTIR measurement

The ATR-FTIR spectra of compounds A, B, 5LIO-1-Cm-3,2-HOPOBn, 5LIO-1-Cm-3,2-HOPO, 5LIO-(Me-3,2-HOPO), UO_2_(NO_3_)_2_·6H_2_O, and the UO_2_-5LIO-1-Cm-3,2-HOPO complex were measured from 4000 to 400 cm^−1^ on a Bruker VERTX 70 FTIR instrument in the transmittance mode.

### Extended X-ray Absorption Fine Structure (EXAFS)

X-ray absorption spectroscopy measurements were performed at beamline 14 W1 of the Shanghai Synchrotron Radiation Facility with a Si (111) double crystal monochromator in transmission mode for the uranium L_3_-edge spectra. The electron beam energy of the storage ring was 3.5 GeV, and the maximum stored current was approximately 210 mA. Energy calibration was performed using a zirconium foil (~17,998 eV). The sample was measured thrice, and the spectra were averaged. The uranium L_3_-edge EXAFS data were analyzed using the standard procedures in the Demeter program^38^. Double-electron excitations affect the EXAFS signal and can influence the results of the data analysis. Thus, in the uranium L_3_-edge EXAFS experimental spectra, the double-electron excitations were subtracted as a reflection of the data translated to the position in energy of the excitation using the standard procedures in Demeter. Supplementary Figure 4 shows the uranium L_3_-edge EXAFS data before and after subtracting the double-electron excitation in k*-* and R*-* space, in which the features at very low distances (R ≈ 1 Å) clearly improved. The coordination number of the uranyl center is expected to be influenced, while the bond length is subtly influenced by double-electron excitations. Theoretical EXAFS data were calculated using FEFF 9.0^39^. Fitting procedure was performed on the k_3_-weighted FTEXAFS from 2–14 Å^−1^. An R window of 1–4 Å was used for the fitting. The amplitude reduction factor, S_0_^2^ was fixed at 0.83 in the EXAFS fits, and the shifts in the threshold energy, ΔE_0_, were constrained to be the same value for all fitted shells.

### Computational method

First principle calculations based on density functional theory (DFT) were performed by using the Gaussian 09 program^40^ to investigate the structural, binding properties, and the quantitative analyses of molecular surfaces for two types of ligands and two types of ligand-uranyl chelates. For the 5LIO-(Me-3,2-HOPO) and the 5LIO-1-Cm-3,2-HOPO ligands, two monomer fragments containing their foremost structural features were used as the computational models, as shown in Fig. 3a and b. For the ligand-uranyl chelate models, the complete double-deprotonated chelating agent molecules were used. Since it is widely recognized that the penta-coordinate UO_2_^2+^ is the dominant species in aqueous solutions, an additional water molecule was added to supplement the vacant coordination site of the uranyl center, as seen in Fig. 3c and d. Full geometric optimizations for all models were carried out in the liquid phase at the B3LYP-D3/SDD~6–31G(d) level. The hybrid-B3LYP exchange-correlation functional^41,42^ combined with the D3 version of Grimme’s dispersion^43^ were employed. The Stuttgart/Dresden relativistic effective core potential^44^ and associated valence basis set (SDD, 60 core electrons and 32 valence electrons)^45^ were applied for uranyl, while the standard Gaussian-type 6–31G(d) basis sets^46^ were used for other light atoms (C, H, O, and N). The solvation effects were included by using SMD implicit solvent model^47^ with water as the solvent. Harmonic vibrational frequencies were calculated afterwards to confirm that the obtained structures possessed stationary points (stable structures) or saddle points (transition states) on their potential energy surfaces. The binding energies (*E*_b_) between the UO_2_^2+^ cation and the remaining ligands (containing the chelating agent and one water molecule, with two negative charges) in ligand-uranyl chelates were calculated by *E*_b_ = *E*(complex) − E(UO_2_^2+^) − *E*(remaining), where the three terms of the right side of this formula represent the total energies of the chelates, the UO_2_^2+^ cations, and the remaining ligands. All geometries used for calculating these three energy terms maintained the same atomic arrangements as the complex structures. Based on the ground state electron densities of the two single-deprotonated chelator structures (Fig. 4a), the global electrostatic potential (ESP) minima on the van der Waals (VDW) surfaces (isodensity = 0.001 a.u.) and the local negative ESP areas, contributed from the two oxygen atoms, were then derived to evaluate the electrostatic properties of the two ligands. The quantitative analyses of molecular surfaces, RGD, and topology analyses were performed using the MultiWFN program^48^.

In order to support the electrostatic amplification effect more strongly, we performed Morokuma scheme energy decomposition analyses (EDA) based on two simplest but most typical models, as seen in Supplementary Figure 6. In these two models, the side chains of two ligands were removed, allowing us to only focus on the O–U interactions. The structures of Fig. 2a, b were optimized at B3LYP-D3/SDD~6–31G* level in gas phase. Then, the EDA calculations were performed at ZORA-PBE/TZP level in gas phase. According to the Morokuma EDA scheme, the total binding energy (E_b_) between the uranyl cation and the ligand fragments can be decomposed as:

$$E_{\rm{b}} = E\left( {{\rm{elstat}}} \right) + E\left( {{\rm{Pauli}}} \right) + E\left( {{\rm{orb}}} \right),$$

where, the *E*(elstat), *E*(Pauli) and *E*(orb) represent the electrostatic interaction, the Pauli repulsion and the orbital interaction, respectively. The *E*(elstat) and *E*(orb) are positive interactions (with negative values) for the binding and the *E*(Pauli) is the negative interaction (with positive value) for the binding. The EDA results are summarized in Supplementary Figure 6c.

The ∆G value was calculated by the equation (3) and (4), and the G_H_+, G_LHn−1_, G_LH_ values were calculated by theoretical calculation.

3 $$LH_n = LH_{n - 1} + H^ +$$

4 $$\Delta G = G_{{\mathrm{H}}^{\mathrm{ + }}} + G_{{\mathrm{LH}}_{{\mathrm{n - 1}}}} - G_{{\mathrm{LH}}_{\mathrm{n}}}.$$

### Cytotoxicity assays of uranyl and chelating agents

To compare the cytotoxicity among the ligands and determine the optimized concentrations of U(VI) and chelating agents for removal assays, cytotoxicity assays were performed first. Cells in log phase were cultured in a 96-well plate for 24 h, and then 0.1 mL culture medium contained 12.4 μM U(VI) and different concentrations of chelating agents: 20.0, 40.0, 80.0, 160.0, and 320.0 μM. After 48 h, 10.0 μL of CCK-8 was added to every well and the cells were cultured for 1–2 h. The absorbance spectrum of every well was obtained by ELIASA (FilterMax F5). The cell survival rate could be calculated using the formula: OD (Experimental group) / OD (control group) × 100%. Each group of above assays was performed with six parallel samples, and the results are shown in Supplementary Table 4. All the independent experiments were statistically tested with SAS. 320.0 μM was considered to be an acceptable concentration of the chelating agents for further assays. In both assays, only culture medium was added to the wells of the control group.

### In vitro uranium removal assays

For the U(VI) uptake and release assay, exponentially growing NRK-52E cells were cultured for 24 h, and then 12.4 μM U(VI) and 320.0 μM of chelating agents were added to the experimental group, while only 12.4 μM U(VI) was added to the control group. After 48 h cultured with U(VI) and chelators, the cell cultures were washed with PBS, trypsinized, counted, and lysed. For the U(VI) release assay, the NRK-52E cells were cultured for 24 h, followed by the addition of 2.0 mL culture medium contained 12.4 μM U(VI) into the experimental and control groups, and then the culture medium contains uranium was discarded 24 h later. Then 320.0 μM of chelating agent was added to the experimental group, while only culture medium was added to the control group. After 24 h the addition of chelating agents, the cell cultures were washed with PBS, trypsinized, counted, and lysed. Each group was performed with three parallel samples. The U(VI) content of the cells were analyzed by ICP-MS (Thermo Scientific) and converted to ng per 10^6^ cells (Supplementary Table 5 and 6).

### In vivo uranium decorporation assays

For the single dosage group with intraperitoneal (ip) injection, U(VI) solutions (UO_2_(NO_3_)_2_·6H_2_O, 3.0 mg) were prepared as that the standard dosage (0.5 mg ^238^U(VI) kg^−1^) was contained in 20.0 mL of 0.14 M NaCl at pH 4–5; all the chelating agent sample solutions were adjusted to pH 7–8 with 0.1 M NaOH, and molar ratio of all the ligands to U(VI) is 92:1, except for the NaHCO_3_ solution, whose molar ratio to U(VI) is 184:1. The 5LIO-1-Cm-3,2-HOPO (29.0 mg) or 5LIO-(Me-3,2-HOPO) (29.3 mg) was dissolved in 1.0 mL DMSO, 2.0 mL 0.14 M NaCl and a certain volume of 0.1 M NaOH solution, then diluted to 6.0 mL by adding 0.14 M NaCl solution; ZnNa_3_-DTPA (37.4 mg) was firstly dissolved in 3.0 mL 0.14 M NaCl, and then adjusted the pH to 7–8 with 0.1 M NaOH, finally diluted to 6.0 mL by adding 0.14 M NaCl solution; 3 mL of HEDP (7.4 mg) or NaHCO_3_ (6.0 mg) solution was prepared following the procedure of ZnNa_3_-DTPA. For the single dosage group with oral administration, all the chelating agent sample solutions were adjust to pH 7–8 with 0.1 M NaOH, and the molar ratio of all the ligands to U(VI) is 307:1. 5LIO-1-Cm-3,2-HOPO (48.5 mg) or 5LIO-(Me-3,2-HOPO) (48.1 mg) was dissolved in 1.0 mL DMSO, 1.0 mL 0.14 M NaCl and a certain volume of 0.1 M NaOH solution, then diluted to 3.0 mL by adding 0.14 M NaCl solution; ZnNa_3_-DTPA (62.3 mg) was firstly dissolved in 1.0 mL 0.14 M NaCl, and then adjusted the pH to 7–8 with 0.1 M NaOH, finally diluted to 3.0 mL by adding 0.14 M NaCl solution. For multiple dosage groups, 5LIO-1-Cm-3,2-HOPO (58.0 mg, molar ratio to U (VI) is 46:1) was dissolved in 2.0 mL DMSO, 10.0 mL 0.14 M NaCl and a certain volume of 0.1 M NaOH solution, then diluted to 24.0 mL by adding 0.14 M NaCl solution; ZnNa_3_-DTPA (74.8 mg, molar ratio to U(VI) is 46:1) was firstly dissolved in 10.0 mL 0.14 M NaCl, and then adjust the pH to 7–8 with 0.1 M NaOH, finally diluted to 24.0 mL by adding 0.14 M NaCl solution. 5LIO-1-Cm-3,2-HOPO (145.0 mg, molar ratio to U (VI) is 92:1) for the 6 h, 12 h, and 24 h delayed multiple dosage groups was dissolved in 5.0 mL DMSO and diluted to 30.0 mL followed the above method.

Female Kunming mice (84 to 86 days old, 30 ± 4 g body weight) were obtained for the in vivo uranium decorporation assay. These animal assays were approved by the Animal Care and Use Committee of Soochow University and were in accordance with the National Institutes of Health guidelines in its guide for the care and use of laboratory animals.

The single dosage group with ip injection: The first assay was carried out to compare the removal efficiency between 5LIO-1-Cm-3,2-HOPO and ZnNa_3_-DTPA, and the second assay was carried out to compare the removal efficiency between 5LIO-1-Cm-3,2-HOPO and 5LIO-(Me-3,2-HOPO), the third assay was carried out to compare the removal efficiency between HEDP and NaHCO_3_. Experimental and control groups of five mice each were intravenously (iv) injected with 0.2 mL ^238^U(VI) solution and 0.5 mL ligand solution was administered by intraperitoneal (ip) injection promptly after. ^238^U-injected control group were only given 0.5 mL saline. All mice were fed with water and food 4 h after the ^238^U injection. Mice were dissected to obtain the kidneys, femur, liver, spleen and muscle samples. The samples of tissues were digested in aqua regia, and the contents of ^238^U in each were determined by ICP-MS (Thermo Scientific). The results of these assays are listed in Supplementary Table 7–10.

The single dosage group with oral administration: Similarly, two assays were carried out to compare the removal efficiency among 5LIO-1-Cm-3,2-HOPO, 5LIO-(Me-3,2-HOPO), and ZnNa_3_-DTPA. The mice had been fasted for 16 h before administering uranium and chelating agents. Experimental and control groups of five mice each were intravenously (iv) injected with 0.2 mL ^238^U(VI) solution, and 0.5 mL ligand solution was administered by gastric tube promptly after. ^238^U-injected control group were only given 0.5 mL saline. Urine and feces were collected and mice were killed 24 h after the ^238^U injection. Mice were dissected to obtain the kidneys, femur, liver, spleen and muscle samples. The samples of tissues and excreta were digested in aqua regia, and the contents of ^238^U in each were determined by ICP-MS (Thermo Scientific). The results of these assays are listed in Supplementary Table 11 and 12.

The multiple dosage and delayed multiple dosage groups: two assays were carried out to compare the removal efficiency between 5LIO-1-Cm-3,2-HOPO and ZnNa_3_-DTPA with different treated time and dosage, respectively. For the multiple dosage and 1 h multiple dosage groups, the experimental and control groups of five mice were i.v.-injected with 0.2 mL ^238^U(VI) solution. For the multiple dosage group, 0.5 mL ligand solution was given by ip injection at 3 min, 6, 24, 48 h, while the 1 h multiple dosage group was given 0.5 mL ligand solution by ip injection at 1, 7, 25, and 49 h. ^238^U-injected control group were only given 0.5 mL saline. All mice were fed with water and food 4 h after the initial ^238^U injection. Urine and feces were collected and the mice were killed 72 h after the ^238^U injection. Mice were dissected to obtain the kidney and femur samples. For the 6 or 12 or 24 h delayed multiple dosage groups, 0.5 mL ligand solution was given by ip injection at 6, 12, 30, and 54 h after the initial iv injection of U(VI) for the 6 h delayed multiple dosage group, the 12 and 24 h delayed multiple dosage group was given 0.5 mL ligand solution by ip injection at 12, 18, 36, 60 h and 24, 30, 48, and 72 h after the initial iv injection of U(VI), respectively. ^238^U-injected control group were only given 0.5 mL saline. All mice were fed with water and food 4 h after the initial ^238^U injection. Mice were dissected to obtain the kidney, femur, liver, spleen and muscle samples. The samples of tissues were digested in aqua regia, and the contents of ^238^U in each were determined by ICP-MS (Thermo Scientific). These results are listed in Supplementary Table 13 and 14.

### Statistical analysis

All above data were presented as mean T standard deviation (SD), and obtained from at least three independent experiments in each assay. For comparison of means of two groups, a paired-sample T-test for independent-samples was applied with SAS software. *p* < 0.05 was considered to be statistically significant.

### In vitro desorption experiments

The in vitro desorption experiments were designed to demonstrate and compare the thermodynamic possibility of 5LIO-1-Cm-3,2-HOPO and 5LIO-(Me-3,2-HOPO) to desorb the complexed uranyl from HAP to further support the experimental performance of the ligand removing uranium from bones.

U(VI) stock solution was obtained by dissolving 60.0 mg UO_2_(NO_3_)_2_·6H_2_O into 60.0 mL HEPES buffer solution (pH = 7.39). ZnNa_3_-DTPA (22.3 mg) was dissolved in 1.0 mL buffer solution, and 5LIO-1-Cm-3,2-HOPO (16.7 mg) or 5LIO-(Me-3,2-HOPO) (16.7 mg) was dissolved in 1.0 mL DMSO. The sample U-0 contained 2.0 mL U(VI) stock solution and 0.1 mL buffer solution. The samples U-1 and U-2 were obtained by mixing 2.0 mL U(VI) stock solution, 0.1 mL buffer solution, and 2.0 mg HAP together. After 180 min and 360 min, the samples of U-1 and U-2 were filtered and collected, respectively. Sample ZnNa_3_-DTPA-1 was obtained by firstly mixing the 2.0 mL U(VI) stock solution and 2.0 mg HAP. After 180 min, 0.1 mL ZnNa_3_-DTPA was added into the uranyl-HAP solution and shook for 15 min before the samples were collected. Sample ZnNa_3_-DTPA-2 was prepared in a similar manner except that the samples were collected 180 min after the ZnNa_3_-DTPA was added into the uranyl-HAP solutions (Fig. 6h). Similarly, samples 5LIO-1-Cm-3,2-HOPO-1, 5LIO-1-Cm-3,2-HOPO-2, 5LIO-(Me-3,2-HOPO)-1, and 5LIO-(Me-3,2-HOPO)-2 were isolated following the same procedure. All samples were kept shaking on the shaking table until they were collected. All samples were filtered using a 0.22 μm filter membrane. The ^238^U contents were determined by ICP-OES (ThermoFisher Scientific iCAP 7000) (Supplementary Table 15).

### Reporting Summary

Further information on research design is available in the Nature Research Reporting Summary linked to this article.

## Supplementary information

Supplementary Information

Description of Additional Supplementary Files

Supplementary Data 1

Supplementary Data 2

Supplementary Data 3

Reporting Summary

Source Data

## Data Availability

Data supporting the findings of this work are available within the paper and its Supplementary Information files. A reporting summary for this article is available as a Supplementary Information file. The source data (PDB files) containing the information of coordinates of DFT optimized structures of UO_2_-5LIO-(Me-3,2-HOPO) (Fig. 1b), UO_2_-5LIO-1-Cm-3,2-HOPO (Fig. 1d, left), and UO_2_-5LIO-1-Cm-3,2-HOPO (Fig. 1d, right) are provided as Supplementary Data 1–3. The source data underlying Figs. 5a, 5b, 6a, 6b, 6c, 6d, 6f, 6g, and Supplementary Table 10 are provided as a Source Data file.
