# Supplementary material for: A 3,2-Hydroxypyridinone-based Decorporation Agent that Removes Uranium from Bones In Vivo
Source: Nat Commun. 2019 Jun 25;10:2570. doi: 10.1038/s41467-019-10276-z (PMC6592941; doi:10.1038/s41467-019-10276-z)
Supplement: Supplementary file 1 — Supplementary Information [file 41467_2019_10276_MOESM1_ESM.pdf]

Supplementary Information for

A 3,2-Hydroxypyridinone-based Decorporation Agent that Removes Uranium from Bones In Vivo

X. Wang *et al*.

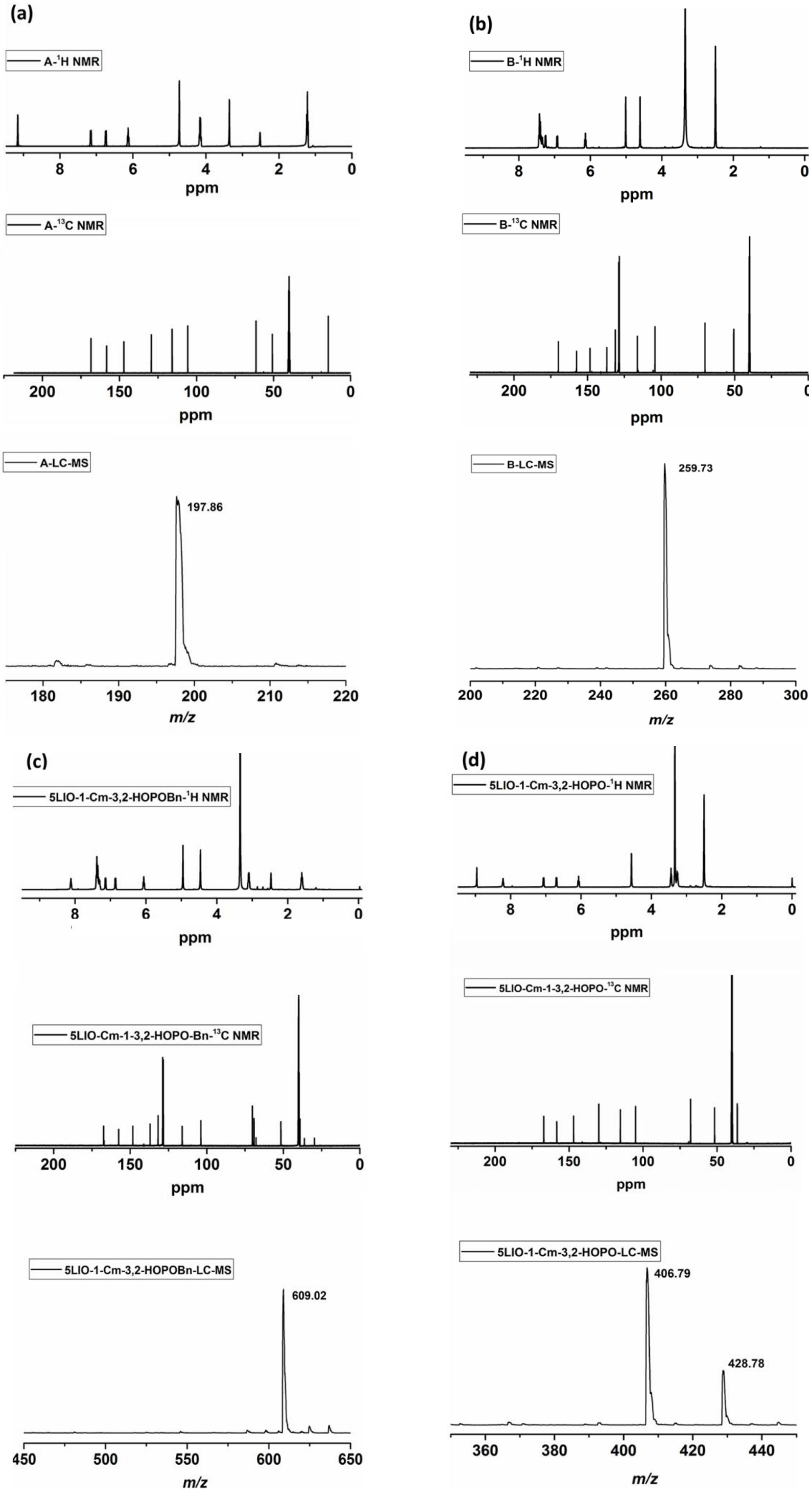

(a)
A-1H NMR
ppm
A-13C NMR
ppm
A-LC-MS
197.86
m/z
(b)
B-1H NMR
ppm
B-13C NMR
ppm
B-LC-MS
259.73
m/z
(c)
5LIO-1-Cm-3,2-HOPOBn-1H NMR
ppm
5LIO-Cm-1-3,2-HOPO-Bn-13C NMR
ppm
5LIO-1-Cm-3,2-HOPOBn-LC-MS
609.02
m/z
(d)
5LIO-1-Cm-3,2-HOPO-1H NMR
ppm
5LIO-Cm-1-3,2-HOPO-13C NMR
ppm
5LIO-1-Cm-3,2-HOPO-LC-MS
406.79
428.78
m/z

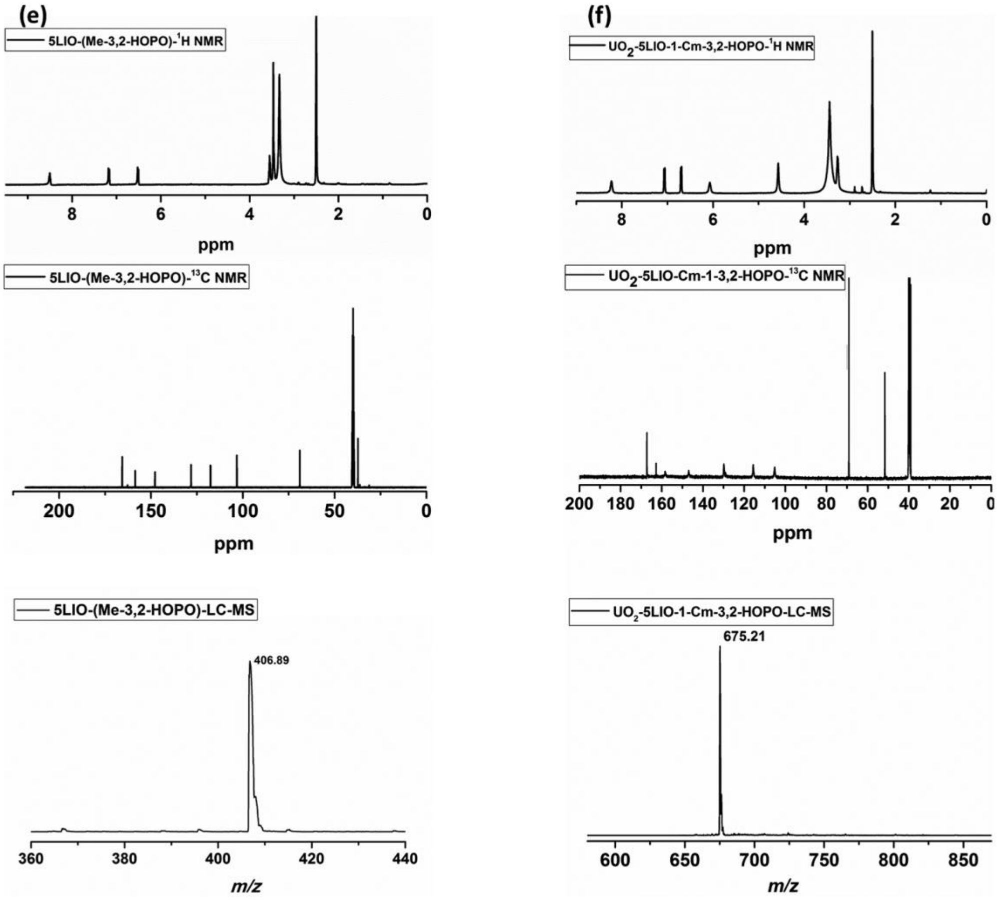

Supplementary Figure 1. NMR and LC-MS characterizations of the synthesized ligands and compounds. (a) - (f), the ^{1}HNMR, ^{13}CNMR, and LC-MS spectra of the compounds of A, B, 5LIO-1-Cm-3,2-HOPOBn, 5LIO-1-Cm-3,2-HOPO, 5LIO-(Me-3,2-HOPO), and the UO_2-5LIO-1-Cm-3,2-HOPO complex.

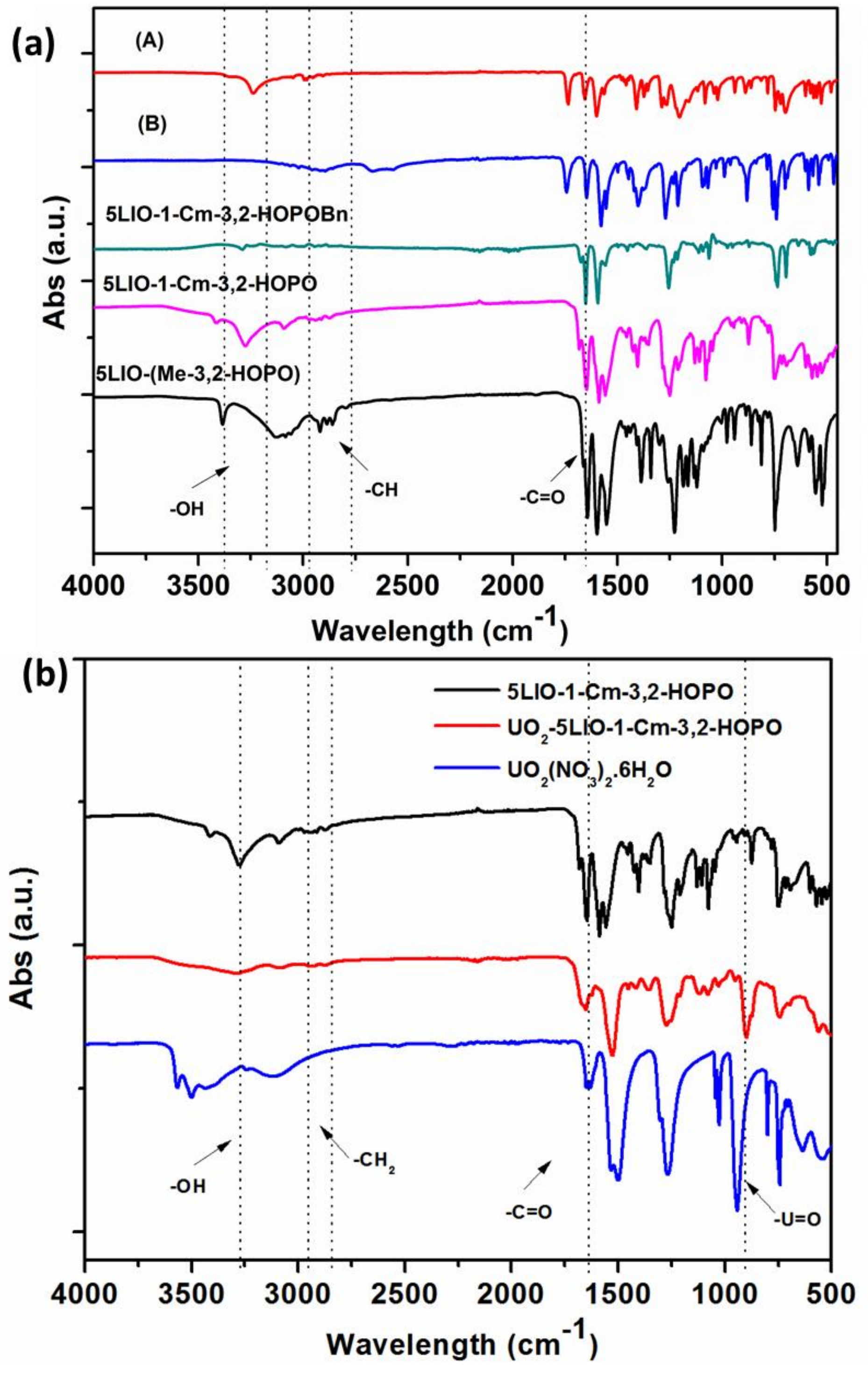

Supplementary Figure 2. ATR-FTIR spectra of the ligands and compounds involved in this study. (a) The ATR-FTIR spectra of the compounds of A, B, 5LIO-1-Cm-3,2-HOPOBn, 5LIO-1-Cm-3,2-HOPO, and 5LIO-(Me-3,2-HOPO); (b) The ATR-FTIR spectra of the compounds of 5LIO-1-Cm-3,2-HOPO, $UO_2(NO_3)$ $6H_2O$, and UO_2-5LIO-1-Cm-3,2-HOPO

complex. (All the data were collected on a Bruker VERTX 70 FTIR instrument in transmittance mode).

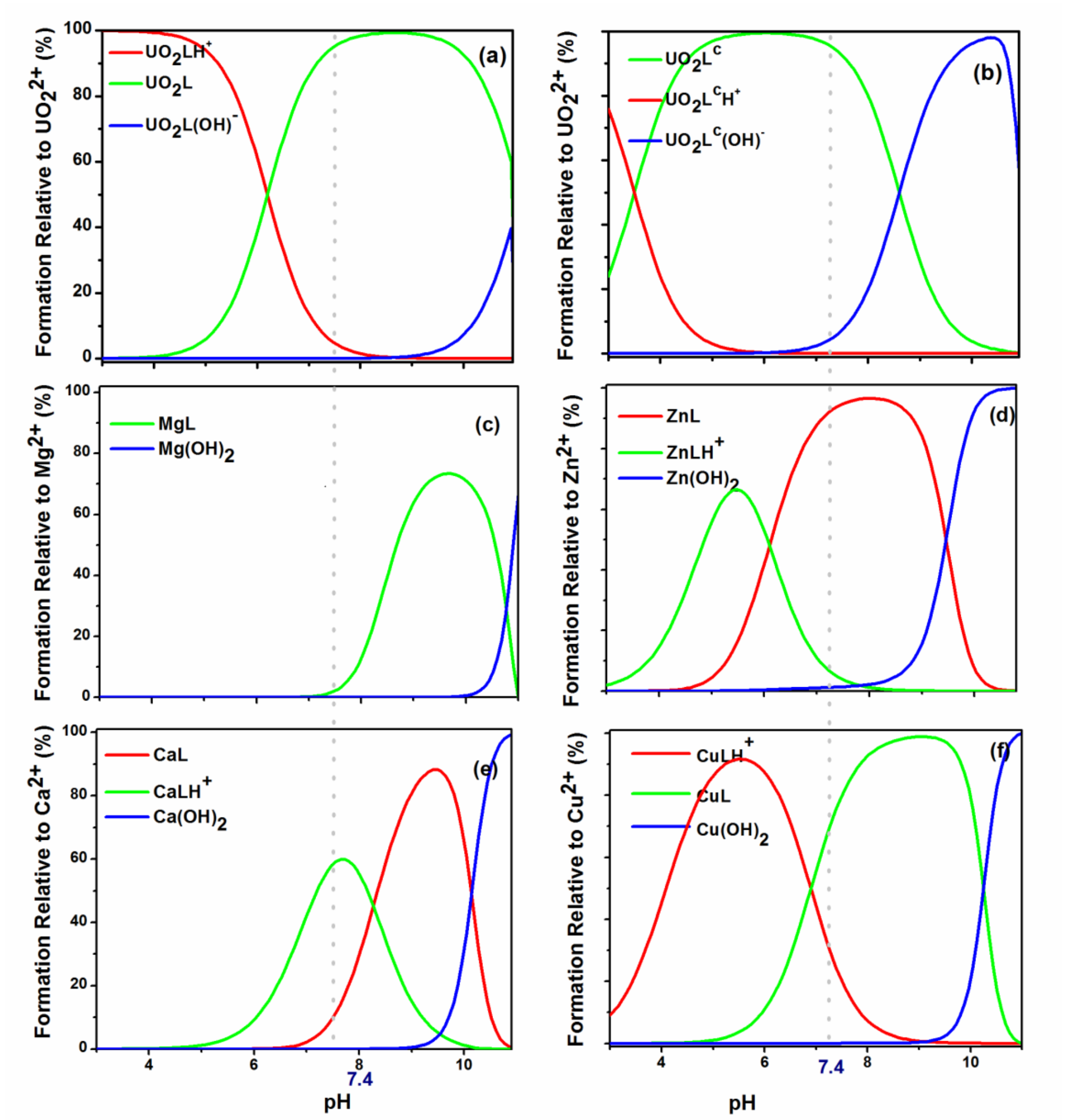

Supplementary Figure 3. The speciation distribution diagrams of metal ions and ligands from pH 3 - 11. Speciation distribution diagrams of (a) UO_2(VI)/L, (b) UO_2(VI)/L^c, (c) Mg(II)/L, (d) Zn(II)/L, (e) Ca(II)/L, and (f) Cu(II)/L systems for solutions containing 1×10^{-4} M of corresponding metal ions and 1×10^{-3} M ligand.

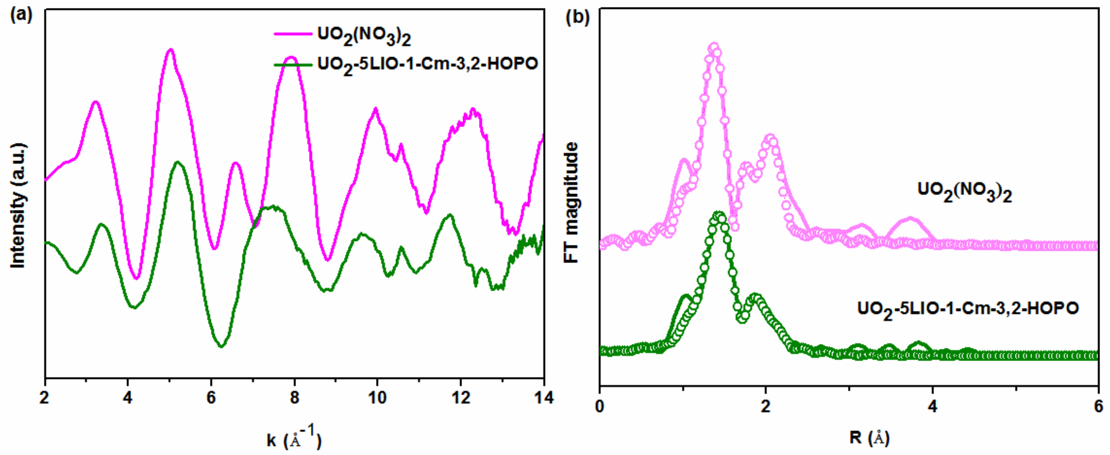

Supplementary Figure 4. Extended X-ray adsorption fine structure (EXAFS) measurement results. (a) EXAFS spectra of $UO_2(NO_3)_2$ and UO_2-5LIO-1-Cm-3,2-HOPO complexes at the U L_3-edge; (b) Fourier transform of the EXAFS spectra of $UO_2(NO_3)_2$ and UO_2-5LIO-1-Cm-3,2-HOPO complexes.

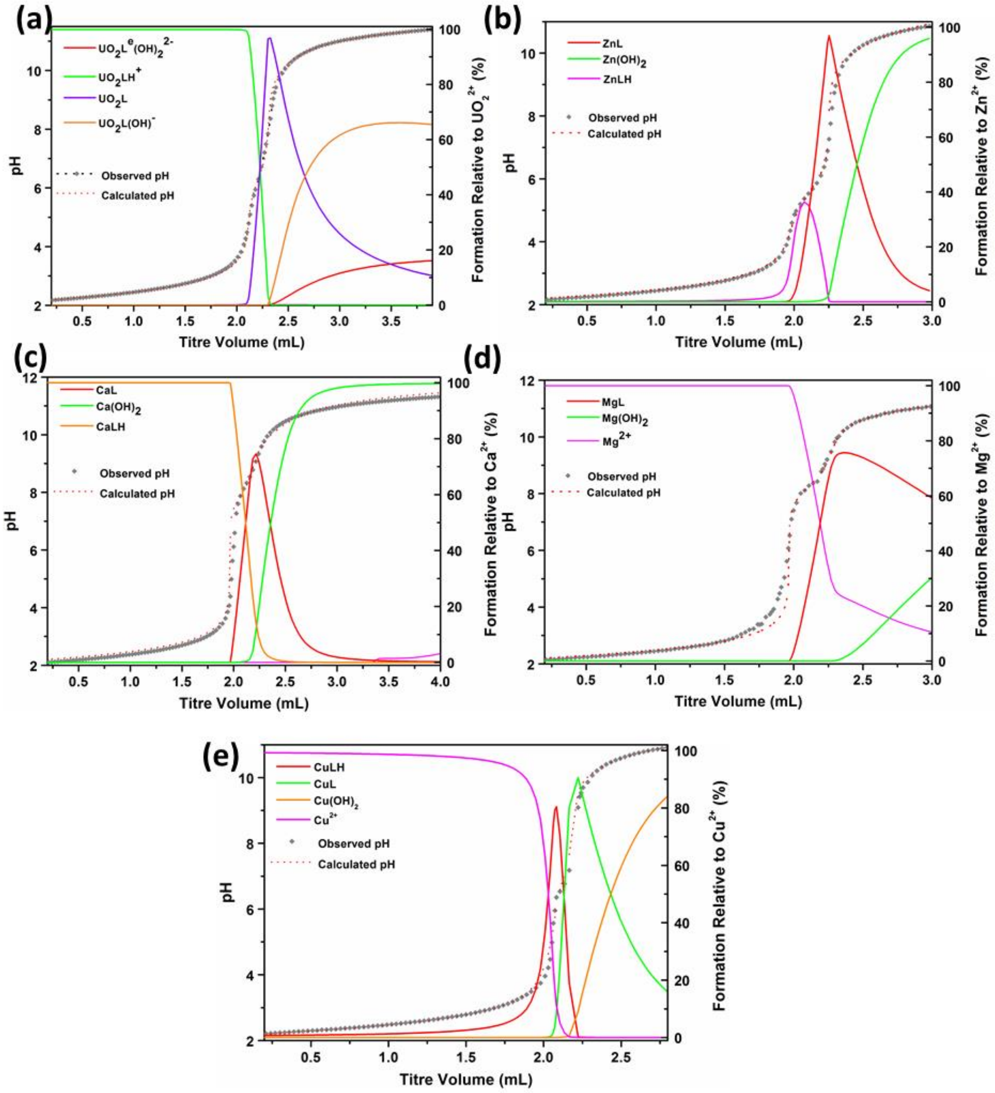

Supplementary Figure 5. The potentiometric data displayed as titre volume vs pH overlaid onto speciation (gray circles and red dots are observed and calculated pH, respectively). (a) UO_2(VI)/L (The contents of the species of uranyl-hydroxide and uranyl-carbonate complexes were too low to show in the figure), (b) Zn(II)/L, (c) Ca(II)/L , (d) Mg(II)/L and (e) Cu(II)/L systems.

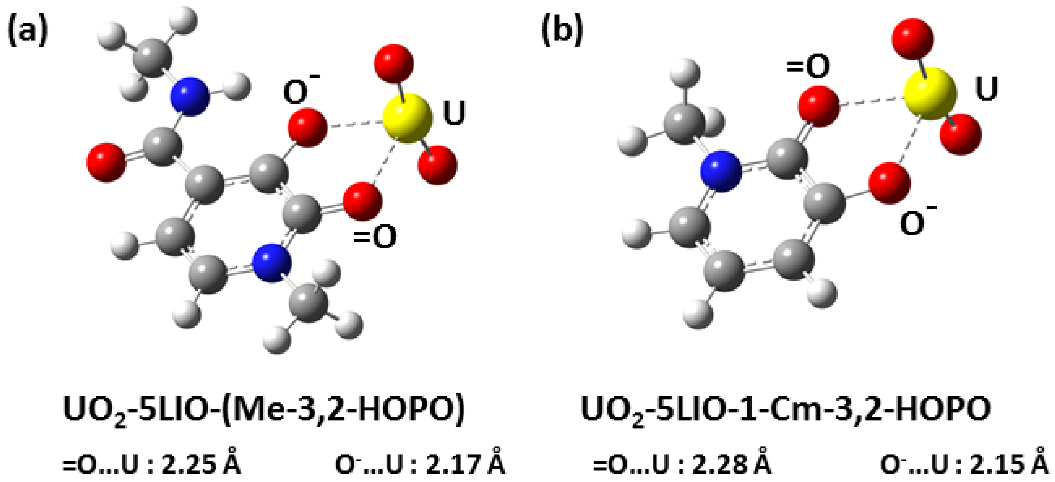

(c)

EDA	E_b (eV)	E(elstat)	E(Pauli)	E(orb)
UO_2-5LIO-(Me-3,2-HOPO)	-17.20	-15.94	8.71	-9.97
UO_2-5LIO-1-Cm-3,2-HOPO	-17.99	-17.10	8.91	-9.80

Supplementary Figure 6. Morokuma scheme energy decomposition analyses (EDA) for UO_2-5LIO-(Me-3,2-HOPO) and UO_2-5LIO-1-Cm-3,2-HOPO. (a) and (b) show the optimized structures of the uranyl cation binding with the UO_2-5LIO-(Me-3,2-HOPO) and UO_2-5LIO-1-Cm-3,2-HOPO fragments; (c) The energy decomposition analyses.

Supplementary Table 1. Formation constants of metal ions and ligands. The formation constants of 5LIO-1-Cm-3,2-HOPO and biological trace elements (Mg^{2+}, Zn^{2+}, Ca^{2+}, and Cu^{2+}), and the formation constants of EDTA and uranyl.

Ligand	Species	m, l, h	Logβ_{mlh}	pM[a]
EDTA	UO_2^{2+}	1, 1, 1	16.4(5)	
		1, 1, 0	11.2(5)	
		1, 1, -1	4.4(7)	
		1, 1, -2	-4.9(8)	
5LIO-1-Cm-3,2-HOPO	Mg^{2+}			4.4(0)
		1, 1, 0	4.6(4)	
		0, 1, -2	-21.0(7)	
5LIO-1-Cm-3,2-HOPO	Zn^{2+}			8.0(7)
		1, 1, 1	15.9(6)	
		1, 1, 0	9.8(7)	
		0, 1, -2	-13.1(7)	
5LIO-1-Cm-3,2-HOPO	Ca^{2+}			4.8(1)
		1, 1, 1	13.9(3)	
		1, 1, 0	5.7(2)	
		0, 1, -2	-19.1(4)	
5LIO-1-Cm-3,2-HOPO	Cu^{2+}			7.9(2)
		1, 1, 1	16.6(5)	
		1, 1, 0	9.7(4)	
		0, 1, -2	-14.9(7)	

[a] pM = -log [M_{free}]; [M] = 10^{-4} M and [L] = 10^{-3} M.

Supplementary Table 2. The refined structural parameters from EXAFS measurement results. EXAFS structural parameters of U-O bonds in $UO_2(NO_3)_2$ and UO_2-5LIO-1-Cm-3,2-HOPO.

Sample	Bond Type[a]	CN[b]	R (Å)[c]	σ^2 (Å^2)[d]	R factor
$UO_2(NO_3)_2$	U-O_{ax}	2	1.77	0.0020	0.018
	U-O_{eq}	5.6 ± 0.6	2.48	0.0084	
UO_2-5LIO-1-Cm-3,2-HOPO	U-O_{ax}	2.2 ± 0.2	1.82	0.0046	0.014
	U-O_{eq}	4.7 ± 0.6	2.41	0.0144	

[a]O_{ax}, O_{eq} refer to coordinated oxygen atoms in the O=U=O axis, or in the equational plane surrounding O=U=O, and distant oxygen atoms from the carbonate ligands, respectively. [b]CN: coordination number (unitless). [c]Error: R ≤ ± 0.02 Å. [d]Debye-Waller factors. Error: $\sigma^2 \leq \pm 0.0008$ Å^2.

Supplementary Table 3. Topological analyses of the ground state electron density of UO_2-5LIO-(Me-3,2-HOPO) and UO_2-5LIO-1-Cm-3,2-HOPO complexes. *V(r)* is the potential energy density at corresponding BCPs. E^{HB} is the hydrogen bond energy calculated by $E^{HB} = V(r) / 2$.

Topological analysis for UO_2-5LIO-(Me-3,2-HOPO)		
Bond Critical Point (BCP)	*V(r)* (Hartree)	E^{HB} (eV)
-NH ··O(pyridine)	-0.02628726650	-0.36
Topological analysis for UO_2-5LIO-1-Cm-3,2-HOPO		
Bond Critical Point (BCP)	*V(r)* (Hartree)	E^{HB} (eV)
-NH ··O(pyridine)	-0.01936586085	-0.27
-NH ··O(uranyl)	-0.00783463886	-0.11
-CH ··O(uranyl)	-0.00381681327	-0.05

Supplementary Table 4. The comprehensive cytotoxicity of uranium and ligands for NRK-52E cells. Dosage-dependent cell growth rate of NRK-52E cells treated with 12.4 μM U(VI) + 5LIO-1-Cm-3,2-HOPO, 12.4 μM U(VI) + 5LIO-(Me-3,2-HOPO) and 12.4 μM U(VI) + $ZnNa_3$-DTPA.

Concentration(μM)	U (VI) + $ZnNa_3$-DTPA Survival Rate (%)	U (VI) + 5LIO-1-Cm-3,2-HOPO Survival Rate (%)	U (VI) + 5LIO-(Me-3,2-HOPO) Survival Rate (%)
20	83.5 ±5.1	86.8 ±1.9	72.1 ±2.4
40	83.0 ±4.1	83.5 ±2.6	55.7 ±1.8
80	82.8 ±5.0	82.7 ±0.5	52.0 ±1.3
160	82.7 ±4.1	80.9 ±2.2	42.6 ±1.7
320	81.1 ±5.0	80.3 ±1.9	41.5 ±0.8

Supplementary Table 5. The uranium removal efficiency of NRK-52E cells. Effects of 5LIO-1-Cm-3,2-HOPO and $ZnNa_3$-DTPA on U(VI) uptake and release in NRK-52E cells.

Group	U(VI) (ng per 10^6 cells)
U(VI) Control	343.9 ± 70.9
U(VI) + $ZnNa_3$-DTPA	301.1 ± 40.0
U(VI) + 5LIO-1-Cm-3,2-HOPO	1.6 ± 0.9

Supplementary Table 6. The uranium removal efficiency of NRK-52E cells. Effects of 5LIO-1-Cm-3,2-HOPO and $ZnNa_3$-DTPA on U(VI) release in NRK-52E cells.

Group	U(VI) (ng per 10^6 cells)
U(VI) Control	33.7 ± 2.2
U(VI) + $ZnNa_3$-DTPA	26.1 ± 2.8
U(VI) + 5LIO-1-Cm-3,2-HOPO	8.2 ± 4.1

Supplementary Table 7. The uranium removal efficiency of $ZnNa_3$-DTPA and 5LIO-1-Cm-3,2-HOPO with single dosage ip injection. U(VI) retention in kidneys and femurs, all values reported as (μg per g), mice were ip injected with ligands (193 μmol kg^{-1}, molar ratio 92:1) after the iv injection of 0.5 mg U(VI) kg^{-1} and then were killed 24 h later.

Tissue Type	U(VI) + NS	U(VI) +$ZnNa_3$- DTPA	U(VI) + 5LIO-1-Cm-3,2-HOPO
Kidneys	6.72 ± 0.72	5.34 ± 2.21	1.16 ± 0.18
Femurs	2.64 ± 0.36	2.40 ± 0.59	1.61 ± 0.16

Supplementary Table 8. The uranium removal efficiency of 5LIO-(Me-3,2-HOPO) and 5LIO-1-Cm-3,2-HOPO with single dosage ip injection. U(VI) retention in kidneys and femurs, liver & spleen & muscle, all values reported as (μg per g), mice were ip injected with ligands (193 μmol kg^{-1}, molar ratio 92:1) after the iv injection of 0.5 mg U(VI) kg^{-1} and then were killed 24 h later.

Tissue Type	U(VI) + NS	U(VI) + 5LIO-(Me-3,2-HOPO)	U(VI)+5LIO-1-Cm-3,2-HOPO
Kidneys	14.11 ± 1.53	2.52 ± 0.51	1.86 ± 0.69
Femurs	4.63 ± 0.58	4.26 ± 0.41	2.41 ± 0.77
Liver & spleen & muscle	0.94 ± 0.14	1.05 ± 0.52	0.82 ± 0.52

Supplementary Table 9. Excretion of U(VI) from urine and feces, as percentages. Mice were ip injected (193 μmol kg^{-1}, molar ratio 92:1) or oral administered (644 μmol kg^{-1}, molar ratio 307:1) with ligand after the iv injection of U(VI) 0.5 mg U(VI) kg^{-1} and then were killed 24 h later (the initial iv injection of ^{238}U is 85.0 μg).

Excretion Source	Urine + Feces (μg)	Urine + Feces (%)
U(VI) + NS (Single dosage group: ip injection)	29.79	35.05
U(VI) + 5LIO-(Me-3,2-HOPO) (Single dosage group: ip injection)	65.25	76.77
U(VI) + 5LIO-1-Cm-3,2-HOPO (Single dosage group: ip injection)	73.84	86.87
U(VI) + 5LIO-(Me-3,2-HOPO) (Single dosage group: oral administration)	45.74	53.81
U(VI) + 5LIO-1-Cm-3,2-HOPO (Single dosage group: oral administration)	67.06	78.89

Supplementary Table 10. The uranium removal efficiency of $ZnNa_3$-DTPA, HEDP, and $NaHCO_3$ with single dosage ip injection. U(VI) retention in kidneys and femurs, liver & spleen & muscle, all values reported as (μg per g), mice were ip injected with ligands (193 μmol kg^{-1}, molar ratio 92:1 for $ZnNa_3$- DTPA and HEDP; 386 μmol kg^{-1}, molar ratio 184:1 for $NaHCO_3$) after the iv injection of 0.5 mg U(VI) kg^{-1} and then were killed 24 h later.

Tissue Type	U(VI) + NS	U(VI) + $ZnNa_3$-DTPA	U(VI) + HEDP	U(VI)+ $NaHCO_3$
Kidneys	8.54 ±1.22	6.86 ±0.69	5.86 ±0.67	8.65 ±3.97
Femurs	5.63 ±1.39	5.85 ±1.08	4.65 ±1.34	4.97 ±0.60
Liver& spleen& muscle	1.21 ±0.74	2.33 ±1.11	0.67 ±0.24	1.47 ±0.97

Supplementary Table 11. The uranium removal efficiency of $ZnNa_3$-DTPA and 5LIO-1-Cm-3,2-HOPO with single dosage oral administration. U(VI) retention in kidneys and femurs, all values reported as (μg per g), mice were oral administered (644 μmol kg^{-1}, molar ratio 307:1) with ligand after the iv injection of 0.5 mg U(VI) kg^{-1} and then were killed 24 h later.

Tissue Type	U(VI) + NS	U(VI) + $ZnNa_3$-DTPA	U(VI) + 5LIO-1-Cm-3,2-HOPO
Kidneys	13.43 ± 1.83	12.43 ± 2.56	5.62 ± 2.21
Femurs	9.74 ± 1.35	8.90 ± 0.85	5.89 ± 0.60

Supplementary Table 12. The uranium removal efficiency of 5LIO-(Me-3,2-HOPO) and 5LIO-1-Cm-3,2-HOPO with single dosage oral administration. U(VI) retention in kidneys, femurs, and Liver & spleen & muscle, all values reported as (μg per g), mice were oral administered (644 μmol kg^{-1}, molar ratio 307:1) with ligand after the iv injection of 0.5 mg U(VI) kg^{-1} and then were killed 24 h later.

Tissue Type	U(VI) + NS	U(VI) + 5LIO-(Me-3,2-HOPO)	U(VI) + 5LIO-1-Cm-3,2-HOPO
Kidneys	14.11 ± 1.53	5.19 ± 1.34	4.49 ± 0.91
Femurs	4.63 ± 0.58	4.47 ± 1.00	3.22 ± 0.59
Liver & spleen & muscle	0.94 ± 0.14	0.75 ± 0.36	0.97 ± 0.30

Supplementary Table 13. The uranium removal efficiency of $ZnNa_3$-DTPA and 5LIO-1-Cm-3,2-HOPO with multiple dosage ip injection. U(VI) retention in kidneys and femurs, all values reported as (μg per g), mice were given the ligand by ip (97 μmol kg^{-1}, molar ratio 46:1) injection at 3 min, 6 h, 24 h, and 48 h (multiple-dose group) or at 1 h, 7 h, 25 h, and 49 h (1 h multiple-dose group) after the iv injection of 0.5 mg U(VI) kg^{-1} and then were killed 72 h later.

Tissue Type	U(VI) + NS	U(VI) + $ZnNa_3$-DTPA	U(VI) + 5LIO-1-Cm-3,2-HOPO	U(VI) + $ZnNa_3$-DTPA (1 h)	U(VI) + 5LIO-1-Cm-3,2-HOPO (1 h)
Kidneys	5.22 ± 1.13	4.55 ± 0.68	1.50 ± 0.39	4.20 ± 1.44	1.52 ± 0.47
Femurs	3.50 ± 0.39	3.17 ± 0.19	1.84 ± 0.26	3.01 ± 0.40	1.75 ± 0.13

Supplementary Table 14. The uranium removal efficiency of 5LIO-1-Cm-3,2-HOPO with delayed multiple dosage ip injection. U(VI) retention in kidneys and femurs, all values reported as (μg per g), mice were given the ligand by ip (193 μmol kg^{-1}, molar ratio 92:1) injection at 6 h, 12 h, 30 h, and 54 h (6 h delayed multiple-dose group), or at 12 h, 18 h, 36 h, and 60 h (12 h delayed multiple-dose group), or at 24 h, 30 h, 48 h, and 72 h (24 h delayed multiple-dose group) after the iv injection of U(VI) (0.5 mg U(VI) kg^{-1}) and were killed 7 d later.

Tissue Type	U(VI) + NS	U(VI) + 5LIO-1-Cm-3,2-HOPO (6 h Delayed)	U(VI) + 5LIO-1-Cm-3,2-HOPO (12 h Delayed)	U(VI) + 5LIO-1-Cm-3,2-HOPO (24 h Delayed)
Kidneys	4.66 ± 0.84	1.80 ± 0.36	1.75 ± 1.02	1.63 ± 0.09
Femurs	4.22 ± 1.39	2.55 ± 0.46	3.20 ± 0.50	2.92 ± 0.85

Supplementary Table 15. Results of in vitro desorption experiments. U(VI) desorption efficiency from HAP using 5LIO-1-Cm-3,2-HOPO, 5LIO-(Me-3,2-HOPO) or $ZnNa_3$-DTPA.

Samples	Input	Initial U(VI) added (μmol)	Sample collected Time (min)	Final U(VI) concentration in solution (ppm)
U-0	h	4	0	495.1
U-1	i	4	180	8.6
U-2	i	4	360	5.3
$ZnNa_3$-DTPA-1	l	4	180 + 15	15.9
$ZnNa_3$-DTPA-2	m	4	180 + 180	16.7
5LIO-1-Cm-3,2-HOPO -1	l	4	180 + 15	294.3
5LIO-1-Cm-3,2-HOPO -2	m	4	180 + 180	387.0
5LIO-(Me-3,2-HOPO) -1	l	4	180 + 15	60.9
5LIO-(Me-3,2-HOPO) -2	m	4	180 + 180	90.5

h: the mixture of 2 mL U(VI) stock solution and 0.1 mL buffer solution;
i: the sample U-1 and U-2 were obtained by mixing 2 mL U(VI) stock solution, 0.1 mL buffer solution, and 2 mg HAP together. Then the solutions were filtered and collected 180 min and 360 min after the addition U(VI), respectively;
l: the samples were obtained following this procedure: 2 mg of HAP was firstly added to 2 mL of U(VI) stock solution, shook for 180 min; then 0.1 mL of $ZnNa_3$-DTPA/5LIO-1-Cm-3,2-HOPO/5LIO-(Me-3,2-HOPO) stock solution was added to the HAP/U(VI) solution and collected 15 min later;
m: the samples were obtained following this procedure: 2 mg of HAP was firstly added to 2 mL of U(VI) stock solution, shook for 180 min; then 0.1 mL of $ZnNa_3$-DTPA/5LIO-1-Cm-3,2-HOPO/5LIO-(Me-3,2-HOPO) stock solution was added to the HAP/U(VI) solution and collected 180 min later.
