# Supplementary material for: A 3,2-Hydroxypyridinone-based Decorporation Agent that Removes Uranium from Bones In Vivo
Source: Nat Commun. 2019 Jun 25;10:2570. doi: 10.1038/s41467-019-10276-z (PMC6592941; doi:10.1038/s41467-019-10276-z)
Supplement: Supplementary file 2 — Description of Additional Supplementary Files [file 41467_2019_10276_MOESM2_ESM.docx]

**Description of Additional Supplementary Files**

File Name: Supplementary Data 1

Description: The information of coordinates of DFT optimized structure of UO2-5LIO-(Me-3,2-HOPO) in Figure 1b.

File Name: Supplementary Data 2

Description: The information of coordinates of DFT optimized structure of UO2-5LIO-1-Cm-3,2-HOPO in Figure 1d, left.

File Name: Supplementary Data 3

Description: The information of coordinates of DFT optimized structure of UO2-5LIO-1-Cm-3,2-HOPO in Figure 1d, right.
