# Supplementary material for: A 3,2-Hydroxypyridinone-based Decorporation Agent that Removes Uranium from Bones In Vivo
Source: Nat Commun. 2019 Jun 25;10:2570. doi: 10.1038/s41467-019-10276-z (PMC6592941; doi:10.1038/s41467-019-10276-z)
Supplement: Supplementary file 6 — Reporting Summary [file 41467_2019_10276_MOESM6_ESM.pdf]

nature research

Corresponding author(s): Shuao Wang, Juan Diwu, and Ruhong Zhou

Last updated by author(s): Mar 29, 2019

Reporting Summary

Nature Research wishes to improve the reproducibility of the work that we publish. This form provides structure for consistency and transparency in reporting. For further information on Nature Research policies, see Authors & Referees and the Editorial Policy Checklist.

Statistics

For all statistical analyses, confirm that the following items are present in the figure legend, table legend, main text, or Methods section.

n/a	Confirmed	
☐	☒	The exact sample size (*n*) for each experimental group/condition, given as a discrete number and unit of measurement
☐	☒	A statement on whether measurements were taken from distinct samples or whether the same sample was measured repeatedly
☐	☒	The statistical test(s) used AND whether they are one- or two-sided<br>*Only common tests should be described solely by name; describe more complex techniques in the Methods section.*
☒	☐	A description of all covariates tested
☐	☒	A description of any assumptions or corrections, such as tests of normality and adjustment for multiple comparisons
☐	☒	A full description of the statistical parameters including central tendency (e.g. means) or other basic estimates (e.g. regression coefficient) AND variation (e.g. standard deviation) or associated estimates of uncertainty (e.g. confidence intervals)
☒	☐	For null hypothesis testing, the test statistic (e.g. *F*, *t*, *r*) with confidence intervals, effect sizes, degrees of freedom and *P* value noted<br>*Give P values as exact values whenever suitable.*
☒	☐	For Bayesian analysis, information on the choice of priors and Markov chain Monte Carlo settings
☒	☐	For hierarchical and complex designs, identification of the appropriate level for tests and full reporting of outcomes
☒	☐	Estimates of effect sizes (e.g. Cohen's *d*, Pearson's *r*), indicating how they were calculated

Our web collection on statistics for biologists contains articles on many of the points above.

Software and code

Policy information about availability of computer code

Data collection	First principle calculations based on density functional theory (DFT) were performed by using the Gaussian 09 program; The quantitative analyses of molecular surfaces, RGD, and topology analyses were performed using the MultiWFN program;
Data analysis	The program GLEE was used to obtain values of the E° and slope; Hyperquad 2013 was used to determine the formation constants ; Hyss 2014 was used to calculate the species distribution of 5LIO-1-Cm-3,2-HOPO and metal ions; Theoretical EXAFS data were calculated using FEFF 9.0; A paired-sample T-test for independent-samples was applied with SAS software.

For manuscripts utilizing custom algorithms or software that are central to the research but not yet described in published literature, software must be made available to editors/reviewers. We strongly encourage code deposition in a community repository (e.g. GitHub). See the Nature Research guidelines for submitting code & software for further information.

Data

Policy information about availability of data

All manuscripts must include a data availability statement. This statement should provide the following information, where applicable:

- Accession codes, unique identifiers, or web links for publicly available datasets
- A list of figures that have associated raw data
- A description of any restrictions on data availability

Data supporting the findings of this work are available within the paper and its Supplementary Information files. A reporting summary for this article is available as a Supplementary Information file. The source data (PDB files) containing the information of coordinates of DFT optimized structures of UO2-5LIO-(Me-3,2-HOPO) (Figure 1b), UO2-5LIO-1-Cm-3,2-HOPO (Figure 1d, left), and UO2-5LIO-1-Cm-3,2-HOPO (Figure 1d, right) are provided as Supplementary Datasets 1-3.

Field-specific reporting

Please select the one below that is the best fit for your research. If you are not sure, read the appropriate sections before making your selection.

☒ Life sciences ☐ Behavioural & social sciences ☐ Ecological, evolutionary & environmental sciences

For a reference copy of the document with all sections, see nature.com/documents/nr-reporting-summary-flat.pdf

Life sciences study design

All studies must disclose on these points even when the disclosure is negative.

Sample size	All the biodata were obtained from at least three independent experiments in each assay. The CCK-8 assays were performed with six parallel samples, in vitro uranium removal assays were performed with three parallel samples, and in vivo uranium removal assays were performed with five parallel samples. For comparison of means of two groups, a paired-sample T-test for independent-samples was applied with SAS software. $p < 0.05$ was considered to be statistically significant.
Data exclusions	No data were excluded from the analyses.
Replication	All the experimental data can be repeated.
Randomization	All the mice used in this paper were grouping randomly.
Blinding	N/A

Reporting for specific materials, systems and methods

We require information from authors about some types of materials, experimental systems and methods used in many studies. Here, indicate whether each material, system or method listed is relevant to your study. If you are not sure if a list item applies to your research, read the appropriate section before selecting a response.

Materials & experimental systems

n/a	Involved in the study
☒	☐ Antibodies
☐	☒ Eukaryotic cell lines
☒	☐ Palaeontology
☐	☒ Animals and other organisms
☒	☐ Human research participants
☒	☐ Clinical data

Methods

n/a	Involved in the study
☒	☐ ChIP-seq
☒	☐ Flow cytometry
☒	☐ MRI-based neuroimaging

Eukaryotic cell lines

Policy information about cell lines

Cell line source(s)	The NRK-52E cell line (organism: kidney, rat, ATCC® CRL-1571™) was purchased from Maisha Bio-Technology, Ltd.
Authentication	The NRK-52E cell line was used as received and the cell morphology is similar with the image from ATCC database.
Mycoplasma contamination	The cell line was uncontaminated and not tested for mycoplasma contamination.
Commonly misidentified lines (See ICLAC register)	The NRK-52E cell line is the only cell line used in our study.

Animals and other organisms

Policy information about studies involving animals; ARRIVE guidelines recommended for reporting animal research

Laboratory animals	Female Kunming mice (84 to 86 days old, 30 ± 3 g body weight) were obtained for the in vivo uranium decorporation assay.
Wild animals	This study did not involve wild animals.
Field-collected samples	This study did not involve samples collected from the field.

Ethics oversight

These animal assays were approved by the Animal Care and Use Committee of Soochow University and were in accordance with the National Institutes of Health guidelines in its guide for the care and use of laboratory animals.

Note that full information on the approval of the study protocol must also be provided in the manuscript.
